# Terpenoid Accumulation and Terpene Synthase Gene Expression Are Coordinately Modulated in Calabrian Pine (*Pinus nigra* subsp. *laricio*) Following Infestation by the Pine Processionary Moth (*Thaumetopoea pityocampa*)

**DOI:** 10.3390/ijms27219775

**Published:** 2026-10-30

**Authors:** Enrica Alicandri, Bartolomeo Sebastiani, Anna Rita Paolacci, Stefano Covino, Agostino Sorgonà, Francesco Manti, Elvira Castiglione, Carmelo Peter Bonsignore, Maurizio Badiani, Mario Ciaffi

**Affiliations:** 1 Dipartimento per la Innovazione nei Sistemi Biologici, Agroalimentari e Forestali, Università della Tuscia, Via S. Camillo De Lellis, s.n.c., I-01100 Viterbo, Italy; enrica.alicandri@unitus.it or e.alicandri@arsial.it (E.A.); arpaolacci@unitus.it (A.R.P.); stefano.covino@unitus.it (S.C.); 2 Dipartimento di Chimica, Biologia e Biotecnologie, Università di Perugia, Via Elce di Sotto 8, I-06123 Perugia, Italy; bartolomeo.sebastiani@unipg.it; 3 Dipartimento di Agraria, Università Mediterranea di Reggio Calabria, Loc. Feo di Vito, I-89129 Reggio Calabria, Italy; asorgona@unirc.it (A.S.); mbadiani@unirc.it (M.B.); 4 Dipartimento di Architettura e Design, Università Mediterranea di Reggio Calabria, Salita Melissari, I-89124 Reggio Calabria, Italy; francesco.manti@unirc.it (F.M.); elvira.castiglione@hotmail.it (E.C.); cbonsignore@unirc.it (C.P.B.)

**Keywords:** biotic stress, Calabrian pine, diterpene resin acids, herbivory, monoterpenoids, oleoresin, specialized metabolism, terpene synthases

## Abstract

Calabrian pine (*Pinus nigra* subsp. *laricio*) is an ecologically and economically important Mediterranean conifer increasingly exposed to the pine processionary moth (PPM; *Thaumetopoea pityocampa*). We investigated temporal differences in needle terpene metabolism between saplings maintained in a control enclosure and saplings exposed to prolonged PPM infestation in a separate, otherwise identical enclosure. Three-year-old saplings underwent semi-natural infestation for 195 days, and needles were sampled at 0, 21, 84, and 195 days post-infestation. Solvent-extractable monoterpenoids and diterpene resin acids (DRAs) were quantified through gas chromatography–mass spectrometry, while the transcript abundances of mono- and diterpene synthase (MTPS and DTPS, respectively) genes were analyzed through quantitative real-time PCR. During the whole experimental period, the terpenoid blend remained unchanged, qualitatively speaking, in both control and PPM-infested saplings, whereas the two experimental groups showed marked time-dependent quantitative differences among each other in terms of individual compound and transcript abundances. In PPM-infested plants, β-pinene, α-pinene, α-terpineol, and (−)-β-phellandrene accumulated mainly at 84 and 195 days, with changes in their putatively associated *Pnl MTPS* transcripts accompanying or preceding metabolite accumulation. Conversely, δ-3-carene and *Pnl MTPS3* transcript abundance declined strongly. Abietane-type DRAs and dehydroabietic acid accumulated at later stages, together with higher *Pnl DTPS1* transcript abundance. Multivariate analyses showed earlier divergence of TPS transcript profiles than metabolite profiles. Thus, Calabrian pine saplings infested by PPM exhibit selective, temporally structured reprogramming of mono- and diterpenoid pathways, rather than a generalized terpenoid induction.

## 1. Introduction

Conifers are long-lived forest trees that must preserve growth and reproduction while maintaining protection against recurrent abiotic and biotic pressures. Their defense strategy relies on constitutive barriers and inducible responses operating across tissues and over different time scales, which allow trees to respond to feeding insects, pathogenic fungi, wounding, and environmental stress [1,2,3]. In Pinaceae, oleoresin is one of the most distinctive and effective defense systems because it acts both as a physical barrier, sealing wounds and entrapping attackers, and as a chemically active matrix rich in bioactive terpenoids [4,5,6]. Such a defense system is particularly relevant under ongoing global change because warming and drought may alter insect pressure, host susceptibility, resin flow, and the balance between preformed and inducible chemical protection [2,4].

The terpenoid fraction of conifer oleoresin includes volatile monoterpenes, semi-volatile sesquiterpenes, and poorly volatile diterpene resin acids (DRAs), which differ in mobility, persistence, and biological function [5,6]. Monoterpenes may act as toxic or deterrent compounds against insects but can also serve as host location cues or pheromone precursors for specialized herbivores and bark beetles [3,4,6,7]. DRAs contribute strongly to resin viscosity, wound sealing, and antimicrobial activity and may influence the success of insect-associated fungi, particularly in bark beetle systems [5,8,9]. The relative importance of these compound classes is therefore expected to depend on the attacker, target tissue, response timing, and degree of specialization of the insect–microbe complex involved [2,10,11,12].

Terpenoid diversity in conifers is generated mainly by the terpene synthase (TPS) gene family. In gymnosperms, most specialized mono-, sesqui-, and diterpene synthases belong to the TPS-d subfamily, whose expansion and functional diversification have contributed substantially to the chemical diversity of *Pinus* spp. and related genera [13,14]. Monoterpene synthases convert geranyl diphosphate into C10 olefins and alcohols, whereas diterpene synthases convert geranylgeranyl diphosphate into diterpene olefins that are subsequently oxidized, mainly by CYP720B cytochrome P450 monooxygenases, to form DRAs [4,5,9]. Many conifer TPS enzymes are multiproduct enzymes, and several terpenoids may originate from overlapping or convergent enzymatic routes; recent work on plant TPSs further emphasizes that product promiscuity and enzyme architecture can be as important as primary sequence similarity in shaping terpene chemodiversity [15]. Consequently, transcript abundance alone may not reliably predict chemical output, and integrated analyses of TPS expression and metabolite accumulation are required to interpret inducible terpenoid responses [16,17,18].

Evidence from conifer–insect systems indicates that induced terpenoid responses are highly specific rather than uniform; they are also dynamic, tissue- and species-specific, and dependent on plant ontogeny and the type of damage or elicitor applied [8,18,19,20,21]. Chewing herbivores and defoliators provide a particularly relevant framework for studying such specificity; in *Pinus pinaster* and *P. radiata*, feeding by the pine weevil *Hylobius abietis* and the pine processionary moth (PPM) *Thaumetopoea pityocampa* induces different chemical responses depending on host species, attacked tissue, and the ecological context of the plant–insect interaction [12]. Resin acid induction in pine seedlings is also compartmentalized and herbivore-specific: stem feeding by *H. abietis* strongly increases stem resin acids, whereas needle feeding by *T. pityocampa* produces weaker or different local and systemic effects depending on pine species [11]. The apparent absence or weakness of induced responses after PPM defoliation in *P. pinaster*, *P. nigra,* and *P. sylvestris* further shows that pine responses to this specialist defoliator cannot be assumed a priori and may depend on the temporal, tissue, and chemical scale analyzed [22].

Terpenoids also influence plant–insect interactions before and during herbivore attack by acting as information-bearing compounds. Female PPM adults perceive components of pine volatile blends, and host selection is affected by the qualitative and quantitative composition of needle monoterpenes [23,24]. Pine volatile bouquets can influence oviposition choice, and selected monoterpenes, including limonene and β-pinene, have been reported to reduce egg laying under field or semi-field conditions [25,26]. A review of PPM-volatile interactions highlighted several compounds able to influence PPM detection, feeding, oviposition, or pheromone-mediated attraction, including limonene, β-pinene, and bornyl acetate [27]. Host plant phenotype can also affect PPM oviposition and egg survival, indicating that chemical cues act together with plant morphological traits and microenvironmental conditions [28].

The ecological and phytosanitary relevance of PPM is increasing across the Mediterranean region. Larvae feed gregariously on pine needles and can cause severe defoliation, growth reduction, and tree weakening, while their urticating hairs represent an important hazard for humans and other mammals in recreational, urban, and peri-urban forests [25,26,29]. PPM population dynamics and activity are influenced by environmental conditions and habitat characteristics [30], while recent analyses indicate that PPM incidence and infestation risk are shaped by warmer winters, stand structure, and landscape-level factors, making PPM an important concern for forest and public health management [29,31,32].

Black pine (*Pinus nigra* J.F. Arnold) is widely distributed in southern Europe and Asia Minor, and its subspecies *laricio* [(Poiret) Maire], commonly known as Calabrian pine, is a key forest component in southern Italy and Corsica [33,34]. Our previous work established the molecular and biochemical framework underlying terpene-based defense in Calabrian pine. Foti et al. [35] showed that in Calabrian pine stands naturally exposed to PPM, volatile terpenoid emissions changed during the larval trophic season, with bornyl acetate being identified as one of the volatile compounds most consistently associated with infestation. A phylogeny-guided analysis of *Pinus* TPSs clarified the evolutionary relationships and functional diversification of the TPS-d subfamily and supported the identification of Calabrian pine TPS sequences representative of major gymnosperm TPS groups [13]. Four full-length cDNAs coding for diterpene synthases (*Pnl DTPS1*–*Pnl DTPS4*) were then isolated, and their tissue-specific expression patterns were compared with the distribution of major DRAs in Calabrian pine organs [16]. In parallel, seven full-length transcripts coding for one 2-methyl-3-buten-2-ol synthase/monoterpene synthase and six monoterpene synthases (*Pnl MBOS1* and *Pnl MTPS2*–*Pnl MTPS7*) were characterized, and their putative product relationships were evaluated against monoterpene profiles in the same plant system [17].

Building on our previous studies, in the present work, we monitored potted Calabrian pine saplings maintained under mesh-covered conditions and followed during a 195-day semi-natural PPM infestation time-course, integrating qRT-PCR analysis of *Pnl MBOS/MTPS* and *Pnl DTPS* genes with GC-MS profiling of the corresponding mono- and diterpenoids. By combining transcript and metabolite data across successive stages of larval development, the present study aimed at exploring coordinated and compound-specific changes in terpene metabolism during PPM infestation in a non-model Mediterranean conifer such as *P. nigra* subsp. *laricio*.

## 2. Results

### 2.1. Terpenoid Profiles and TPS Transcript Abundance Before PPM Infestation

Figure 1A and Table S1 show, respectively, the monoterpenoids detected in Calabrian pine needles and their relative abundances before the start of PPM infestation (T0). GC-MS analysis detected 14 solvent-extractable monoterpenoids, of which (−)-β-phellandrene and α-pinene were the most abundant. Limonene and β-pinene were also major components, whereas terpinolene and α-thujene represented intermediate fractions. The remaining compounds, including α-terpineol, sabinene, β-myrcene, δ-3-carene, camphene, tricyclene, α-fenchene, and bornyl acetate, were individually present at lower abundances.

At T0, transcript abundances of *Pnl MBOS1* and of the six *Pnl MTPS* genes (*Pnl MTPS2*–*Pnl MTPS7*) varied markedly among genes (Figure 1B; Table S1). *Pnl MBOS1* showed the highest relative expression, followed by *Pnl MTPS6*. *Pnl MTPS5* showed an intermediate expression level, followed by *Pnl MTPS2* and *Pnl MTPS7*, whereas *Pnl MTPS4* and *Pnl MTPS3* were expressed at comparatively lower levels.

As shown in Table 1, only 5 of the 14 monoterpenoids could be putatively associated with the *Pnl MTPS* genes analyzed here. The remaining nine monoterpenoids detected in Calabrian pine needles were not assigned to a specific Calabrian pine MTPS, whereas *Pnl MBOS1* was putatively associated with 2-methyl-3-buten-2-ol synthase activity; this product was not quantified in the present workflow (see Section 4.3, below).

The basal monoterpenoid and MTPS profiles showed qualitative correspondence for some putative pairs, particularly the (−)-β-phellandrene/*Pnl MTPS6* association and the α-pinene/*Pnl MTPS5* and α-pinene/*Pnl MTPS7* associations.

Before PPM infestation, the diterpenoid fraction consisted of nine DRAs (Figure 2A; Table S2). Seven compounds were non-dehydrogenated DRAs, namely abietic, isopimaric, levopimaric, neoabietic, palustric, pimaric, and sandaracopimaric acids, whereas dehydroabietic acid and one unidentified dehydroisomer of abietic acid represented the dehydrogenated fraction. Dehydroabietic and abietic acids were predominant, followed by isopimaric acid, whereas palustric, levopimaric, sandaracopimaric, neoabietic, and pimaric acids, together with the unidentified abietic acid dehydroisomer, were minor components. Neutral diterpene olefins and aldehydic intermediates were below the detection limits of the analytical methods employed.

At T0, transcript abundance of the *Pnl DTPS* genes varied markedly (Figure 2B; Table S2). *Pnl DTPS1* and *Pnl DTPS2* showed the highest relative expression levels, with the former only moderately higher than the latter. *Pnl DTPS4* was expressed at an intermediate level, whereas *Pnl DTPS3* showed the lowest expression, comparatively speaking. According to the pathway-level associations summarized in Table 1, seven of the nine DRAs detected in needles could be linked to the analyzed *Pnl DTPS* genes. In contrast, dehydroabietic acid and the unidentified abietic acid dehydroisomer were not putatively assigned to a specific *Pnl DTPS*, and no DRA product was assigned to *Pnl DTPS2*.

The correspondence between basal *Pnl DTPS* transcript abundance and DRA accumulation was only partial. This is consistent with the fact that DTPSs generate diterpene olefin precursors, which undergo subsequent oxidation to form DRAs, and with the unresolved product relationship of *Pnl DTPS2*. The clearest qualitative agreement involved *Pnl DTPS1*, whose high basal transcript abundance was consistent with the predominance of abietane-related DRAs in the needle DRA pool.

### 2.2. Time-Course of Monoterpenoid Accumulation and Pnl MBOS/MTPS Transcript Abundance During PPM Infestation

Baseline contrasts were examined for every metabolite and TPS transcript (Tables S3–S6). Four variables differed between the groups assigned to the two enclosures before nest attachment: α-thujene and bornyl acetate concentrations and *Pnl MTPS5* and *Pnl MTPS6* transcript abundances were lower in the group assigned to subsequent infestation; all DRA and *Pnl DTPS* baseline contrasts and all remaining monoterpenoid and *Pnl MBOS/MTPS* baseline contrasts were not significant. Across the 195-day time-course, the same 14 monoterpenoids detected at T0 were present at all subsequent sampling times in both control and PPM-infested saplings, with no additional compounds detected (Table S3). All monoterpenoids showed significant Treatment × Time interactions in linear models fitted to log2-transformed concentrations (Table S3). In control needles, monoterpenoid levels and *Pnl MTPS* transcript abundances were generally stable over time, whereas saplings in the PPM-infested enclosure showed compound- and gene-specific temporal differences from controls (Figure 3 and Figure 4; Tables S3 and S4).

Among monoterpenoids putatively associated with specific *Pnl MTPS* genes (Table 1), the β-pinene/*Pnl MTPS4* pair showed the clearest early and progressive divergence from controls (Figure 3; Tables S3 and S4). α-terpineol and α-pinene increased in PPM-infested plants from 84 DPI onward. *Pnl MTPS2* preceded its putative metabolite, showing higher transcript abundance already at 21 DPI; among the two transcripts putatively associated with α-pinene, *Pnl MTPS5* increased from 84 DPI onward, whereas *Pnl MTPS7* diverged mainly at 195 DPI.

The (−)-β-phellandrene/*Pnl MTPS6* pair showed a more delayed response, with both metabolite and transcript becoming higher in PPM-infested plants at 195 DPI (Figure 3; Tables S3 and S4). In contrast, δ-3-carene and its putatively associated transcript *Pnl MTPS3* were the only assigned monoterpenoid/MTPS pair showing consistently lower values in the PPM-infested group from 21 DPI onward (Figure 3; Tables S3 and S4).

The nine monoterpenoids without a specific *Pnl MTPS* assignment also contributed substantially to the time-dependent reorganization of the monoterpenoid pool (Figure 4; Table S3). Several compounds, including α-fenchene, camphene, sabinene, β-myrcene, terpinolene, and bornyl acetate, were higher in PPM-infested plants from 84 DPI onward and remained higher at 195 DPI. Tricyclene and limonene showed a more delayed response, with clear divergence from controls only at the final sampling time. α-thujene was lower in PPM-infested plants at 21 and 84 DPI but became markedly higher than in controls at 195 DPI.

The *Pnl MBOS1* response is shown separately in Figure 5 because its putatively associated product, namely 2-methyl-3-buten-2-ol (MBO), was not quantified in the solvent-extractable GC-MS workflow used here. Among the monoterpenoid-associated genes examined here, *Pnl MBOS1* showed the earliest and largest difference among the two experimental groups and remained higher in the PPM-infested plants throughout the time-course (Table S4).

Overall, monoterpenoid metabolism showed temporally structured differences between the control and PPM-infested enclosures. The early phase was characterized by higher *Pnl MBOS1* transcript abundance, higher β-pinene/*Pnl MTPS4* values, and lower δ-3-carene/*Pnl MTPS3* values in the PPM-infested group. Later, a broader quantitative increase in the monoterpenoid pool occurred, including compounds with and without putative association with MTPS genes. This partial correspondence between transcript and metabolite profiles supports coordinated but compound-specific modulation of the monoterpenoid pathway during PPM infestation.

### 2.3. Time-Course of Diterpene Resin Acid Accumulation and Pnl DTPS Transcript Abundance During PPM Infestation

As observed for monoterpenoids, the same nine DRAs detected at T0 were present at all subsequent sampling times in both control and PPM-infested saplings, with no additional compounds detected (Table S5). Most DRAs showed significant Treatment × Time interactions in linear models fitted to log2-transformed concentrations, except for sandaracopimaric acid and the abietic acid dehydroisomer (Table S5). In control needles, DRA levels and *Pnl DTPS* transcript abundances were generally stable over time, whereas saplings in the PPM-infested enclosure showed branch- and gene-specific temporal differences from controls (Figure 6, Figure 7 and Figure 8; Tables S5 and S6). Among DRAs with putative pathway-level *DTPS* assignments, the clearest divergence involved the abietane-type DRA group associated with *Pnl DTPS1*, which was higher in PPM-infested plants from 84 DPI onward and remained higher at 195 DPI (Figure 6; Tables S5 and S6). This response was particularly evident for palustric and levopimaric acids, which showed marked increases at 84 DPI. Neoabietic and abietic acids also increased, with abietic acid accounting for a large fraction of the absolute increase in the DRA pool. *Pnl DTPS1* transcript abundance increased in PPM-infested saplings, with a detectable difference already at 21 DPI and much stronger divergence at 84 and 195 DPI. This temporal pattern was consistent with a predominance of the abietane-type branch in the PPM-infested group, although the association remains at the pathway level because DTPS enzymes generate diterpene olefin precursors rather than DRAs directly.

Compared with the abietane-type group, the pimarane-type DRA group putatively associated with *Pnl DTPS3* showed a different temporal pattern (Figure 6; Tables S5 and S6). Sandaracopimaric acid remained statistically unchanged throughout the time-course, with no significant Treatment × Time interaction or time-specific treatment contrast. Isopimaric acid showed only a limited treatment-dependent response, with a significant reduction in PPM-infested plants at 195 DPI. *Pnl DTPS3* transcript abundance did not differ between treatments at 21 or 84 DPI but became lower in PPM-infested plants at 195 DPI. Thus, unlike the abietane-type group, the *Pnl DTPS3*-associated pimarane-type branch did not show a broad increase in the PPM-infested group.

Pimaric acid and the transcript abundance of its putatively associated gene, namely *Pnl DTPS4*, were both lower in the PPM-infested group (Figure 6; Tables S5 and S6). Pimaric acid was lower in PPM-infested plants at 21 DPI and again at 195 DPI, whereas the treatment contrast was not significant at 84 DPI. *Pnl DTPS4* transcript abundance was already strongly reduced at 21 DPI and remained lower in PPM-infested plants at both later sampling times. These patterns show that the pimaric-acid-associated branch differed from the abietane-type branch, with persistently lower transcript abundance and a partially corresponding decrease of the associated DRA in the PPM-infested group.

*Pnl DTPS2* was analyzed separately because no DRA product could be assigned to this gene in the a priori association framework (Figure 7; Table S6). Its transcript abundance showed a strong and sustained reduction in PPM-infested plants from 21 DPI onward, closely resembling the response observed for *Pnl DTPS4* (Figure 6). However, this transcriptional response could not be linked to a defined DRA in the present dataset.

DRAs without a specific putative assignment to a *Pnl DTPS* showed contrasting profiles (Figure 8; Table S5). Dehydroabietic acid, the most abundant DRA at T0, increased strongly in PPM-infested plants at 84 and 195 DPI, broadly paralleling the abietane-type DRA group (Figure 6). In contrast, the unidentified dehydroisomer of abietic acid remained unchanged throughout the experimental infestation and did not show significant Treatment, Time, or Treatment × Time effects (Table S5).

Overall, diterpenoid metabolism showed temporally structured differences between the control and PPM-infested enclosures. The group difference was dominated by higher levels of abietane-related DRAs and dehydroabietic acid at 84 and 195 DPI in the PPM-infested enclosure, accompanied by higher *Pnl DTPS1* transcript abundance. In parallel, *Pnl DTPS2* and *Pnl DTPS4* transcript abundances were markedly lower from the early phase of the time-course, and pimaric acid showed a partial reduction consistent with the behavior of *Pnl DTPS4*. The weaker or absent response of sandaracopimaric and isopimaric acids, together with late repression of *Pnl DTPS3*, further indicated that the two experimental groups did not differ uniformly across all diterpenoid branches. These results support coordinated but branch-specific modulation of DRA metabolism and *Pnl DTPS* transcript abundances.

### 2.4. Multivariate Overview of Metabolic and Transcriptional Profiles During the PPM-Infestation Experiment

To integrate the metabolite and transcript responses described above, multivariate analyses were performed on the complete metabolite and *TPS* transcript matrices (Figure 9). In the metabolite PCA, the first two principal components together explained 79.4% of the total variance (Table S7). The PCA scores plot showed that PC1 provided the main axis of separation and reflected the progressive shift of PPM-infested saplings away from controls during the time-course. Samples collected at 0 DPI largely overlapped, whereas PPM-infested samples moved progressively toward positive PC1 values at 84 and 195 DPI, consistent with the broad quantitative increase in monoterpenoid and DRA pools observed in the univariate analyses (Figure 9A).

Variables contributing positively to metabolites PC1 included most monoterpenoids that were higher at later sampling times in the PPM-infested group, such as α-pinene, β-pinene, α-fenchene, camphene, sabinene, β-myrcene, terpinolene, α-terpineol, and bornyl acetate, together with the abietane-related DRAs palustric, levopimaric, abietic, and neoabietic acids and the unassigned dehydroabietic acid (Table S7). In contrast, δ-3-carene and, to a lesser extent, isopimaric acid showed negative PC1 loadings, consistent with their distinct or reduced profiles in PPM-infested plants. PC2 captured additional temporal structure: α-thujene and pimaric acid showed among the strongest positive loadings, whereas isopimaric acid, the abietic acid dehydroisomer, and several abietane-related DRAs showed negative loadings (Table S7). This axis contributed to the separation of PPM-infested samples collected at 84 and 195 DPI (Figure 9A). Thus, the metabolite PCA summarized the condition- and time-related pattern as a coordinated but compound-specific reorganization of the terpenoid profile rather than a uniform increase in all compounds.

Compared with the metabolites PCA, the *TPS* transcript-based PCA showed an even clearer separation between treatments and sampling times (Figure 9B). The first two principal components together accounted for 85.5% of the total variance (Table S7). As in the metabolite-based PCA, the 0 DPI samples from the two treatment-assigned groups largely overlapped among each other, whereas PPM-infested samples progressively diverged from controls after infestation. Transcript PC1 mainly reflected the opposing regulation of transcriptionally activated and repressed TPS genes. Strong positive PC1 loadings were observed for *Pnl MBOS1*, *Pnl MTPS2*, *Pnl MTPS4*, *Pnl MTPS5*, and *Pnl DTPS1*, whereas negative loadings were observed for *Pnl MTPS3*, *Pnl DTPS2*, *Pnl DTPS3*, and *Pnl DTPS4*. PC2 further resolved the temporal progression of PPM-infested samples, separating the 21-, 84-, and 195-DPI groups (Figure 9B). *Pnl MTPS6* and *Pnl MTPS7* showed the largest positive PC2 loadings (0.497 and 0.492, respectively; Table S7). Consistent with this pattern, both transcripts were lower in PPM-infested than in control plants at 21 DPI; *Pnl MTPS6* remained lower at 84 DPI, whereas both transcripts were higher in PPM-infested plants at 195 DPI (Table S4). This multivariate pattern was consistent with simultaneous activation of selected mono- and diterpene synthase transcripts and repression of other *TPS* branches during PPM infestation.

Hierarchical clustering provided an independent visualization of the same multivariate structure (Figures S5 and S6). In the metabolite heatmap, late PPM-infested samples clustered apart from most control and pre-infestation samples, mainly because of the increased abundance of several monoterpenoids, abietane-related DRAs, and dehydroabietic acid, together with the contrasting behavior of δ-3-carene, pimaric acid, and selected pimarane-type DRAs. Compared with the metabolites heatmap, separation between PPM-infested and control samples was sharper in the transcript heatmap, particularly from 21 DPI onward, reflecting early transcriptional activation of *Pnl MBOS1*, *Pnl MTPS2*, *Pnl MTPS4,* and *Pnl DTPS1* and concomitant repression of *Pnl MTPS3*, *Pnl DTPS2,* and *Pnl DTPS4*. These clustering patterns support the view that transcriptional reprogramming preceded or accompanied later and broader metabolic divergence.

PERMANOVA indicated that both datasets were structured by experimental Condition, Time, and their interaction (Table 2). In the metabolites matrix, Treatment, Time, and Treatment × Time explained 25.2%, 30.8%, and 31.8% of the total multivariate variation, respectively, and all three model terms had *p* < 0.001 within this experiment. In the *TPS* transcript matrix, Treatment and Treatment × Time accounted for 38.5% and 37.8% of the variation, respectively, whereas Time accounted for 20.8%; all three model terms again had *p* < 0.001 within this experiment. The larger Condition and Condition × Time components in the transcripts dataset described a particularly strong temporal divergence of transcript profiles between the two enclosures. Time-specific PERMANOVA contrasts showed no separation between treatment-assigned groups at 0 DPI and progressively larger condition contrast effect sizes at later times, as reflected by increasing R^2^ and pseudo-F values. However, the exact permutation framework with three plant-level biological samples per condition limited the resolution of time-specific *p* values (Table S8).

The progressive divergence between control and PPM-infested plants was also evident from normalized Euclidean distances between treatment centroids (Figure 9C). For metabolite profiles, the normalized distance was low at 0 DPI, increased modestly at 21 DPI and then rose sharply at 84 and 195 DPI. Compared with metabolite profiles, *TPS* transcript divergence was already more pronounced at 21 DPI and increased further at 84 and 195 DPI. This difference in timing was consistent with the univariate results in which several transcript responses were already detectable during the early phase of infestation, whereas broad metabolite accumulation became most evident at 84 and 195 DPI.

Pearson correlations describe covariation between *TPS* transcript abundances and putatively associated metabolites within the a priori assignment framework defined in Table 1. Among monoterpenoid-associated genes, all product-level associations were positive and significant after false discovery rate correction (Table S9). The strongest correlations involved *Pnl MTPS3* with δ-3-carene, *Pnl MTPS5* with α-pinene, *Pnl MTPS4* with β-pinene, and *Pnl MTPS2* with α-terpineol; *Pnl MTPS6* was strongly correlated with (−)-β-phellandrene, and *Pnl MTPS7* showed a weaker but still significant association with α-pinene. Among diterpenoid-related genes, *Pnl DTPS1* was strongly correlated with all four abietane-type DRAs, whereas *Pnl DTPS3* showed no significant correlation with sandaracopimaric acid and a weaker positive correlation with isopimaric acid. *Pnl DTPS4* was positively correlated with pimaric acid. No correlation was tested for *Pnl MBOS1* because MBO was not quantified in the present analytical workflow and no DRA was assigned to *Pnl DTPS2*. Because these analyses pooled both experimental groups and all four sampling times, shared condition and time trends may contribute to the coefficients; the correlations therefore do not demonstrate direct gene–product relationships.

Overall, multivariate analysis showed coordinated, time-dependent, and pathway-specific divergences of the complete terpenoid and *TPS* transcript profiles between the two experimental enclosures. The transcript matrix diverged early and strongly from the control condition, whereas the metabolite matrix showed a more gradual divergence that became most pronounced during later infestation stages. Together with the correlation analysis, these results provide an integrated view of the biochemical and transcriptional responses described in Section 2.2 and Section 2.3, showing coordinated but selective differences in monoterpenoid and diterpenoid pathways rather than generalized activation of all TPS-associated terpenoid branches.

## 3. Discussion

### 3.1. Selective Terpene Reprogramming During PPM Infestation

The present exploratory study identifies selective temporal differences in terpene metabolism between control saplings and saplings exposed to PPM rather than a generalized increase in all terpenoid products. *T. pityocampa* is a specialized gregarious folivore whose larvae feed collectively on pine needles over an extended period rather than causing a single localized wound. The semi-natural experiment reported here therefore represents a prolonged and temporally structured herbivore challenge in which feeding intensity and its physiological consequences may have changed as infestation progressed. This biological context is consistent with the coexistence of early transcriptional responses and later changes in internal terpenoid pools. Thus, induction, repression, and stability coexisted within the same needle tissue.

These findings extend the biochemical and molecular framework previously established for Calabrian pine in which *Pnl MBOS/MTPS* and *Pnl DTPS* genes were identified and related to basal mono- and diterpenoid profiles [13,16,17]. The present work moves beyond basal characterization to a semi-natural herbivore challenge by integrating internal metabolite pools and *TPS* transcript abundances across successive stages of PPM infestation. It also complements the results of Foti et al. [35], which showed that naturally infested Calabrian pine stands differ in volatile terpenoid emissions. Here, the focus was on internal solvent-extractable needle pools and their transcriptional counterparts, providing a molecular and biochemical view of needle reprogramming during PPM infestation.

The present results are consistent with a broader literature showing that PPM-induced terpene responses are heterogeneous rather than uniformly directional. Previous studies reported increases, decreases or limited changes in pine terpene-related traits depending on host species, tissue, defense trait, and experimental context [12,22,39,40]. Recent work on *P. halepensis* further supports this compound-specific view by linking post-defoliation foliage chemistry to reduced PPM larval performance, with changes involving bornyl acetate, p-cymene and diterpenoid-related compounds [41]. Our time-course helps reconcile these findings by showing that accumulation, repression, and limited change can occur simultaneously within the same pine–PPM system, depending on the compound and sampling time considered.

The needle internal pools might not necessarily reflect the airborne cues perceived by PPM. Female PPMs respond to emitted pine volatile blends, and compounds such as limonene, β-pinene, β-myrcene, β-phellandrene, terpinolene, and bornyl acetate have been implicated in host selection, oviposition, or feeding behavior [23,24,25,26,27,42]. Several of these compounds also changed in our needle extracts, and bornyl acetate increased in infested needles, as reported in the volatile-emission study of Foti et al. [35]. Although the two datasets are not directly equivalent, this overlap identifies candidate compounds for future volatile emission and behavioral studies.

### 3.2. Compound- and Branch-Specific Modulation of Mono- and Diterpenoid Pathways

The monoterpenoid fraction provided a clear example of compound-specific reprogramming. Among compounds with putative *Pnl MTPS* assignments, the β-pinene/*Pnl MTPS4* module increased early and persistently, α-terpineol and α-pinene accumulated mainly at intermediate or late stages, and the (−)-β-phellandrene/*Pnl MTPS6* module diverged mainly at the final sampling time. The different temporal profiles of *Pnl MTPS5* and *Pnl MTPS7*, both putatively associated with α-pinene, further suggest that induced monoterpenoids were not regulated as a single synchronized group.

Several monoterpenoids without a specific *Pnl MTPS* assignment also increased in response to PPM infestation, indicating that minor constituents of volatile blends might have disproportionate effects on insect perception and behavior [27]. By contrast, the coordinated repression of δ-3-carene and *Pnl MTPS3* represented an equally important pattern, showing that the response included downregulation of a specific monoterpenoid branch rather than simple stimulation of monoterpene biosynthesis. The monoterpenoid response therefore reflects selective redistribution within the pathway rather than uniform accumulation of all C10 compounds.

The DRA fraction provided a complementary component of the response to PPM. DRAs differ from monoterpenoids in volatility, storage and defensive function, contributing to resin properties and chemical protection in conifers [2,4,5]. In Calabrian pine needles, abietane-type DRAs were higher in the PPM-infested group, in parallel with increased *Pnl DTPS1* transcript abundance. By contrast, the pimarane-type branch and other *Pnl DTPS* transcripts showed weak, absent or repressive responses.

This branch-specific response is consistent with conifer DRA biosynthesis, in which diterpene synthases form olefin precursors that are subsequently oxidized, mainly by CYP720B enzymes, to resin acids [4,9,38]. Thus, the *Pnl DTPS*-DRA relationships used here should be regarded as pathway-level associations. The late increase of abietane-type DRAs differs from the rapid local resin responses often observed after bark beetle-associated fungal inoculation or stem-feeding injury [8,11], but it shows that a specialist defoliator can be associated with a measurable DRA response in needles when individual compounds are followed over a sufficiently long time-course.

### 3.3. Transcript–Metabolite Coordination and Temporal Organization

In the present study, terpenoid accumulation was compared with the expression of the putatively corresponding TPS genes within an a priori assignment framework. This approach was particularly appropriate for Calabrian pine, a non-model conifer in which our previous studies identified major mono- and diterpene synthase candidates [16,17], although the corresponding enzyme–product relationships remain to be validated directly. The assignments summarized in Table 1 therefore provided a biologically informed framework for interpreting the time-course, while the correlations in Table S9 quantified transcript–metabolite covariation across experimental conditions and sampling times. They are exploratory, may be influenced by shared conditions and time trends, and do not establish direct gene–product relationships. Accordingly, the transcript–metabolite comparisons should not be interpreted as proportional relationships between transcript and metabolite abundance. In addition to uncertainties arising from multiproduct or functionally overlapping enzymes and incomplete gene–product assignments, gene-specific translation and enzyme activity, substrate availability and metabolic flux, product turnover and storage, and post-transcriptional or post-translational regulation can decouple transcript abundance from steady-state metabolite levels.

For monoterpenoids, positive correlations between *Pnl MTPS* transcripts and their putatively associated products describe covariation consistent with, but not proof of, transcriptional coordination of several pathway branches. This was evident not only for several modules showing higher values in the PPM-infested group, such as *Pnl MTPS4*/β-pinene, *Pnl MTPS5*/α-pinene, *Pnl MTPS2*/α-terpineol and *Pnl MTPS6*/(-)-β-phellandrene, but also for the *Pnl MTPS3*/δ-3-carene module, which, instead, showed lower values in these same plants. The latter case clarifies the meaning of the correlations: a positive relationship does not necessarily indicate induction, but rather coordinated changes in the same direction across the dataset.

The diterpenoid dataset was more complex. *Pnl DTPS1* was strongly associated with the abietane-type DRAs assigned to this branch, consistent with their higher concentrations at later sampling times in PPM-infested plants. By contrast, *Pnl DTPS3* showed weak or absent correspondence with pimarane-type DRAs, and *Pnl DTPS4* was correlated with pimaric acid despite the overall lower values of this module in the PPM-infested group. Such partial correspondence is expected because DTPS enzymes produce diterpene olefin precursors rather than DRAs directly, and downstream oxidation contributes substantially to final resin acid composition. Therefore, these correlations are compatible with coordinated pathway regulation but neither distinguish shared condition/time trends from within-group coupling nor demonstrate direct enzymatic production of the measured DRAs.

Two additional cases define the boundaries of interpretation, namely the *Pnl MBOS1/*MBO module, and the case of *Pnl DTPS2*, to which no specific DRA could be associated. These limitations further illustrate the intrinsic complexity of terpene biosynthesis and the uncertainty of sequence-based assignments [15,36,37,38]. Functional assays, as demonstrated in other pine–herbivore systems [18], will therefore be required to validate these assignments.

Several *Pnl TPS* transcripts diverged from controls at 21 DPI. By contrast, the broadest changes in internal monoterpenoid and DRA pools became most evident at 84 and 195 DPI. This temporal offset is compatible with transcriptional modulation preceding or accompanying later metabolite accumulation or depletion; however, sampling time also covaried with larval development and unquantified feeding intensity, so these contributions cannot be separated.

Multivariate analyses describe the same temporal pattern. Normalized Euclidean distances between condition centroids followed the same trend, and PERMANOVA showed that Treatment (the experimental condition), Time and their interaction structured both metabolite and transcript profiles within this experiment. Although time-specific contrasts were interpreted cautiously because exact permutations were limited by the number of plant-level biological samples, increasing condition-contrast effect sizes at later sampling times were consistent with progressive divergence after the pre-infestation stage.

The above temporal organization is consistent with broader evidence that conifers can mount rapid and stage-dependent responses to insect attack. In Scots pine, exposure to sawfly sex pheromones has been shown to prime defense against subsequent egg deposition by *Diprion pini*, and transcriptomic analyses have revealed distinct responses to egg deposition, larval feeding and their combination [43,44]. In grand fir, wound-induced oleoresinosis involves differential transcriptional control of mono-, sesqui- and diterpene synthase genes, with *TPS* transcript accumulation preceding or accompanying subsequent oleoresin formation [45]. Our observations are consistent with this concept for a defoliator under semi-natural conditions and illustrate why a single sampling point may lead to different conclusions depending on the compound or transcript examined and on when sampling is performed.

### 3.4. Herbivore Identity, Target Tissue, and Ecological Interpretation

The responses of Calabrian pine to PPM must also be interpreted in relation to feeding guild and target tissue. PPM is a specialized foliar herbivore of pine and cedar species, whereas many well-characterized conifer defense models involve bark beetles, bark-chewing insects, or associated fungi that directly affect phloem, resin ducts, and wound sites. The observed differences among the two experimental groups of plants are compatible with a foliar response to prolonged defoliation expressed through selective differences in internal monoterpenoid and DRA pools, as well as *TPS* transcript profiles. Such changes may therefore include systemic or distal responses, as well as responses occurring before visible tissue injury occurs, rather than exclusively reflect the chemistry of directly consumed needle tissue.

Recent evidence from *P. brutia* infested by the Mediterranean pine shoot beetle *Tomicus destruens* further supports the importance of tissue context: volatile terpene profiles differed between healthy and infested trees in both phloem and needles, but the compounds associated with infestation differed between tissues [46]. Thus, the time-dependent differences in specific metabolite and transcript profiles observed here are compatible with a tissue- and herbivore-specific response during PPM infestation rather than a reduced version of a bark beetle or wounding response.

### 3.5. Limitations and Future Perspectives

Several limitations should be considered when interpreting the present findings. Most importantly, only one enclosure was used for each experimental condition so that experimental condition and enclosure were not independently replicated, preventing their respective contributions from being statistically separated. Nevertheless, random assignment, matched enclosure conditions, the use of a separate nest collected from a different host tree for each infested sapling, the absence of widespread baseline differences, and the progressive, compound-specific divergence that emerged after larval feeding began support a biological interpretation consistent with PPM exposure. These features do not remove the inferential limitation, but they support treating the observed time-course patterns as biologically informative associations.

A further limitation is that dynamic emission of volatiles, including MBO, was not measured here. Changes in β-pinene, limonene, bornyl acetate, or other compounds should therefore be interpreted as internal metabolic responses rather than direct evidence of altered airborne cues perceived by PPM. Sesquiterpenes and the *TPS* genes involved in their biosynthesis were also not analyzed, consistent with the scope of the molecular and biochemical framework currently available for Calabrian pine, which has focused on monoterpenoids, DRAs, and the corresponding *Pnl MBOS/MTPS* and *Pnl DTPS* genes [16,17].

Functional interpretation also remains limited because the *TPS*–product assignments are inferential: no functional assays were performed to validate enzyme–product relationships, and no bioassays tested consequences for larval performance, oviposition, or natural enemy interactions. Future studies should integrate internal terpene pools and headspace emissions with functional characterization of responsive *TPS* and *CYP720B* genes, quantitative measurements of larval development, biomass and feeding damage, and behavioral or performance bioassays. Such studies would help distinguish exposure duration from larval development and feeding intensity while assessing the ecological consequences of the observed responses.

Because PPM affects Mediterranean forest and public health, validated chemical or molecular markers could ultimately support more selective monitoring and biological control across natural, urban, and peri-urban pine systems [29,31,47].

Even within these limitations, the observed patterns identify terpene branches and candidate compounds for validation in independently replicated PPM experiments, thereby providing a foundation for mechanistic studies of terpene-mediated defense in Calabrian pine.

## 4. Materials and Methods

### 4.1. Plant Material, Experimental Site, and Pine Processionary Moth Infestation

The experiment was carried out under semi-natural conditions at the Calabria Regional Observatory for Biodiversity, Santo Stefano in Aspromonte, Cucullaro locality, Southern Italy (38.172128° N, 15.816535° E; 1156.9 m a.s.l.), within the Aspromonte National Park (Figure S1). The site is characterized by pure stands of Calabrian pine naturally exposed to infestation brought about by PPM.

Twenty-four three-year-old potted Calabrian pine saplings were obtained from the Calabria Regional Forest nursery (Calabria Verde Agency, Catanzaro, Italy). Before the experiment, saplings were selected for uniform size, good health status, and absence of visible symptoms attributable to previous PPM infestation or other biotic damage. Two weeks before experimental infestation, they were randomly assigned to two groups of twelve plants each: one allocated to PPM infestation and one maintained as a non-infested control. The control group was placed in one greenhouse-type enclosure and the infestation-assigned group in a second identical but physically separated enclosure, located approximately 10 m apart; both enclosures were covered with the same 2 mm mesh plastic netting and exposed to open-field environmental conditions (Figure S2A,B). No additional independent control or infested enclosures were available. Plants were watered twice weekly throughout the experiment.

At the end of October 2022, intact PPM larval nests of comparable size and weight were collected from infested pine trees near the experimental site. Each nest originated from a different host tree and was inspected visually to confirm the presence of active larvae and the absence of evident mortality or contamination. A separate nest was attached with plastic ties (Figure S3A) to each sapling assigned to the PPM treatment. At the time of attachment, larvae were considered second instars based on external morphology and were approximately 10–15 mm long (Figure S3B). Larval activity, nest integrity, and visible mortality were monitored throughout the experiment. Larval abundance, larval biomass, needle consumption, and defoliation severity were not quantified.

The infestation experiment lasted from late October 2022 to mid-May 2023 and was designed to follow the natural progression of larval development, from the early stages (second larval stage, with larvae leaving the nests) to the fifth larval stage, when the “procession” was completed, thereby covering the period during which the most intense feeding activity occurs under the environmental conditions of the site.

Establishment of the PPM challenge was evidenced qualitatively by progressive defoliation and the presence of PPM larvae, both already observable 21 days after nest attachment on the saplings of the infested enclosure, whereas no comparable symptoms were observed in control plants. No additional visible symptoms attributable to other biotic agents or abiotic stress were observed in either group during the experimental period.

### 4.2. Needle Sampling and Experimental Design

Needle sampling followed a balanced 2 × 4 sampling scheme, with two experimental conditions (non-infested control enclosure and PPM-infested enclosure) and four sampling times. Three plant-level biological replicates for each condition and time point were adopted. The randomized allocation of individual saplings to sampling times is summarized in Figure S2A, and the experimental timeline is shown in Figure S4. Overall, 24 plant-level biological samples were obtained, each corresponding to needle material collected from a single sapling. These plants represented independent biological sampling units within an enclosure but not independent enclosure-level replicates of the experimental conditions.

Needle sampling was consistently performed between 09:00 and 11:00 h to reduce possible diel variation in transcript abundance and terpene metabolism. The first sampling was performed immediately before PPM nests were attached to saplings assigned to the infestation treatment and was therefore denoted as T0 (0 days post-infestation, DPI). Subsequent samplings were performed at 21, 84, and 195 DPI and were denoted as T21, T84, and T195, respectively (Figure S4). The four sampling times were selected according to the developmental and feeding phases summarized in Figure S4: T0 provided the pre-feeding baseline; T21 was assumed to cover the early feeding phase, run by PPM larvae at the L2–L3 stage; T84 for winter trophic activity, with progressive defoliation; and T195 for the late feeding phase, run by larvae at the L5 stage and preceding the procession. Such spacing over time allowed a prolonged natural feeding period to be sampled while retaining three distinct plant-level biological samples per experimental condition at each destructive sampling time.

At each sampling time, needle samples were collected from three different control saplings and as many different infestation-assigned or infested saplings, yielding three plant-level biological samples per experimental condition. Needle tufts were excised from apparently healthy and undamaged portions of the plants. In infested saplings, where feeding damage and partial defoliation were evident, visibly damaged needles were avoided whenever possible, and only apparently intact needles were collected for subsequent biochemical and molecular analyses. Samples were not pooled across plants; each sampled individual constituted one plant-level biological sampling unit for estimating within-enclosure variability.

Immediately after excision, needle material was frozen in liquid nitrogen, transported to the laboratory under frozen conditions, and stored at −80 °C until extraction of terpenoid metabolites and total RNA. Once a plant had been sampled at a given time point, it was marked and excluded from subsequent samplings to avoid carry-over effects caused by previous sampling wounds. The experiment was therefore treated as an independent sampling time-course rather than a repeated-measures design.

### 4.3. Extraction, GC-MS Analysis, and Quantification of Terpenoids

Monoterpenoid and diterpenoid metabolites were extracted from the same needle samples and analyzed through gas chromatography–mass spectrometry (GC–MS) using an Agilent 7890A gas chromatograph coupled to an Agilent 5975C VL MSD Triple-Axis detector (Agilent Technologies, Santa Clara, CA, USA). The analytical workflows followed validated procedures previously developed for Calabrian pine, with minor adaptations to the present infestation time-course [16,17]. Full details of instrumentation, column characteristics, carrier gas conditions, injector settings, MS interface, and detector parameters are reported in Alicandri et al. [16,17], whereas the essential extraction, acquisition, and quantification steps are summarized below. At each sampling time and for each experimental condition, three plant-level biological samples were processed, and each sample was analyzed in triplicate.

For monoterpenoid profiling, approximately 500 mg of frozen needle material was extracted through sonication in 1 mL of n-pentane at room temperature for 15 min. An aliquot of each extract was transferred to a conical vial and analyzed through fast GC-MS. The GC temperature program started at 45 °C for 1 min, increased to 250 °C at 30 °C min^−1^ and was held for 0.5 min, then increased to 325 °C at 45 °C min^−1^ and held for 5 min. MS acquisition was carried out in full-scan mode for qualitative analyses and in selected-ion monitoring mode for quantification, as described previously [17]. The quantified monoterpenoids corresponded to the solvent-extractable monoterpene fraction detected in Calabrian pine needles; 2-methyl-3-buten-2-ol was not quantified with this workflow because of its high volatility and the lack of a dedicated quantitative volatile emission sampling procedure.

For diterpenoid profiling, needle material was dried in the dark at room temperature, cut into small fragments, and extracted twice with n-hexane/dichloromethane (1:1, *v*/*v*) through sonication at 25 °C for 20 min. The two extracts were pooled, dehydrated through anhydrous Na_2_SO_4_, and stored in the dark at −20 °C until clean-up and derivatization. Before GC-MS analysis, aliquots were purified on graphitized carbon to remove non-terpenoid impurities, dried under a gentle stream of N_2_ and derivatized for 30 min at 65 °C with a 1:1 (*v*/*v*) mixture of N,O-bis(trimethylsilyl)trifluoroacetamide containing 1% trimethylchlorosilane and pyridine. Samples were dried again under N_2_, resuspended in n-hexane, and kept at −20 °C until GC-injection [16]. For diterpene resin acids (DRAs), the GC program started at 90 °C for 2 min, increased to 350 °C at 44.7 °C min^−1^, and was held for 5 min. Quantification focused on the DRA fraction, whereas neutral diterpene olefins and aldehydic intermediates were inspected qualitatively, as in Alicandri et al. [16].

Monoterpenoids and diterpenoids were identified by comparing experimental mass spectra with the NIST08 and Wiley02 libraries and with literature spectra and retention information for conifer terpenoids [9,11,16,17,37,38,48]. Monoterpenoids were quantified using calibration curves prepared with commercial α-pinene (98% purity; catalog no. 147524) and (R)-(+)-limonene (97% purity; catalog no. 183164) standards, whereas DRAs were quantified against an abietic acid standard (catalog no. 00010) after derivatization (Sigma-Aldrich, St. Louis, MO, USA). Concentrations were expressed on a dry weight basis and used for subsequent univariate and multivariate statistical analyses.

For both the analytical workflows described above, blank injections were used to verify the absence of interfering peaks at the retention times of target analytes. Analytical precision and extraction recovery were assessed according to the validation criteria reported previously for the same plant system, including intra- and inter-day variability of spiked samples and recovery estimates from spiked plant material [16,17].

### 4.4. RNA Isolation, cDNA Synthesis, and qRT-PCR Analysis

Total RNA was isolated from the same frozen needle samples used for terpenoid profiling. RNA extraction, concentration assessment, and quality checking followed procedures previously applied to Calabrian pine TPS analyses [16,17]. First-strand cDNA was synthesized from 3 µg of total RNA using the Xpert cDNA Synthesis Kit (GRiSP Research Solutions, Porto, Portugal), following the manufacturer’s instructions.

Transcript abundance was analyzed through quantitative real-time PCR (qRT-PCR) for genes previously isolated and characterized in Calabrian pine [16,17]. The target genes included one 2-methyl-3-buten-2-ol synthase/monoterpene synthase gene, *Pnl MBOS1* (GenBank accession OL689404), six monoterpene synthase genes, *Pnl MTPS2*–*Pnl MTPS7* (OL689405–OL689410), and four diterpene synthase genes, *Pnl DTPS1*–*Pnl DTPS4* (OK245418–OK245421). Primer pairs for all target and reference genes are listed in Table S10.

qRT-PCR reactions were performed using an AriaMx real-time PCR system (Agilent Technologies, Santa Clara, CA, USA) with Fast Q-PCR Master Mix (SMOBIO Technology, Hsinchu, Taiwan). Each reaction was carried out in a final volume of 20 µL containing 1 µL of cDNA template and 150 nM of each forward and reverse primer. No-template and no-reverse-transcription controls were included to verify the absence of reagent contamination, primer dimer artifacts, and genomic DNA carry-over. The amplification program consisted of initial denaturation at 95 °C for 2 min, followed by 40 cycles at 95 °C for 15 s and 56 °C for 1 min, and a final dissociation curve step to confirm amplicon specificity.

Primer design, specificity testing, and amplification efficiency assessment were conducted as reported previously for the same set of Calabrian pine DTPS and MBOS/MTPS genes [16,17]. Only primer pairs producing a single melting peak, no detectable non-specific amplification, and efficiencies within the accepted range were used for expression analysis.

Expression data were normalized using the geometric mean of two reference genes, coding for cyclophilin (*CYP*) or for the uncharacterized protein LOC103705956 (*upLOC*). The use of multiple reference genes followed the normalization strategy described by Vandesompele et al. [49], and reference gene stability was evaluated using the model-based approach implemented in NormFinder v21 for Excel [50], as previously applied in Calabrian pine [16,17]. Raw Ct values were converted into relative quantities using primer-specific amplification efficiencies, and normalized relative expression values were calculated by dividing target gene relative quantities by the geometric mean of the two reference gene quantities.

For each experimental condition and sampling time, the three plant-level biological samples described in Section 4.2 were analyzed independently. Each plant-level biological sample was assayed in three technical qRT-PCR replicates, which were averaged before statistical analysis. The resulting normalized relative expression values were used for the univariate and multivariate analyses described in Section 4.6.

### 4.5. Assignment of Putative TPS–Terpenoid Associations

To facilitate comparison of transcript abundance and metabolite accumulation over the experimental period and to highlight possible coherence among temporal patterns, Table 1 summarizes the putative associations between each *Pnl MBOS/MTPS* or *Pnl DTPS* gene and one or more terpenoid products considered to be derived from the corresponding enzymatic activity. These associations should be considered putative, i.e., literature-based, because no functional characterization of Calabrian pine TPSs, for example, by expressing them as recombinant proteins in heterologous systems and assaying their activity in vitro [9,37], was carried out in the present work. Instead, associations were inferred from the phylogenetic placement and sequence similarity of the isolated *Pnl TPS* genes [13], functional characterization of homologous or closely related terpene synthases from other *Pinus* species [36,37,38], and previous biochemical and molecular characterization of mono- and diterpenoid metabolism in Calabrian pine [16,17].

For monoterpenoids, putative associations were based on characterized MBO and monoterpene synthases from *P. sabiniana*, *P. contorta,* and *P. banksiana* [36,37]. Accordingly, *Pnl MBOS1* was retained as the transcript counterpart of 2-methyl-3-buten-2-ol synthase activity, although 2-methyl-3-buten-2-ol itself was not quantified in the solvent-extractable GC-MS workflow because of its high volatility. The *Pnl MTPS* genes were associated only with monoterpenoids for which a sufficiently supported relationship with functionally characterized pine mono-TPSs was available. No specific TPS–product assignment was therefore made for the remaining monoterpenoids detected in needles.

For diterpenoids, assignments were made at the pathway level rather than as direct enzyme–product relationships because diterpene synthases generate olefin precursors that can be further oxidized to DRAs. Accordingly, *Pnl DTPS1* was associated with abietane-type DRA biosynthesis through levopimaradiene-, abietadiene-, neoabietadiene-, and palustradiene-related precursors; *Pnl DTPS3* was associated with isopimaric and sandaracopimaric acids through the corresponding isopimaradiene and sandaracopimaradiene precursors; and *Pnl DTPS4* was associated with pimaric acid through pimaradiene. No DRA was assigned to *Pnl DTPS2* because the product relationship of the closest functionally tested class-I DTPS candidates remains unresolved [38].

### 4.6. Statistical Analysis

Technical replicates were averaged within each plant-level biological sample before statistical analysis. A single sapling sampled once was used as the plant-level sampling unit. Because different plants were destructively sampled at each time point, plant-level observations were treated as independent for analysis of within-enclosure variation so that no repeated-measures model was fitted. However, the experimental condition was applied at the enclosure level, with only one enclosure per condition; consequently, the present experiment lacked independent treatment-level replication.

All statistical analyses were performed using JMP Pro 15.2.1 procedures (SAS Institute Inc., Cary, NC, USA). Statistical analyses were based on the transformed datasets specified below, whereas graphical summaries display untransformed means ± SEM.

For graphical presentation of basal and time-course profiles, qRT-PCR normalized relative expression values and absolute metabolite concentrations were plotted as untransformed means ± SEM of three plant-level biological samples. For statistical inference, transcript abundance was analyzed using log2-transformed normalized relative expression values, whereas absolute metabolite concentrations were analyzed using log2-transformed values. This common transformation was used to reduce scale-dependent variance, limit the influence of highly abundant compounds or transcripts, and express condition-related differences on a multiplicative scale.

For each transcript and metabolite, an exploratory linear model including Treatment, Time, and the Treatment × Time interaction was fitted; here, Treatment denotes the experimental condition assigned to the two enclosures. Treatment had two levels, saplings in the non-infested control enclosure and saplings in the PPM-infested enclosure, whereas Time had four levels, 0, 21, 84, and 195 DPI. Model-derived pairwise contrasts were used to compare control and infested plants at each time point. When comparisons among all Treatment × Time combinations were required, post hoc tests were performed on model-adjusted means. Families of related pairwise tests were adjusted using the Benjamini–Hochberg false discovery rate procedure [51]. Residual normality and homogeneity of variance were assessed using the Shapiro–Wilk and Levene tests, respectively [52,53].

Relative metabolite composition was calculated separately for monoterpenoids and diterpenoids as the percentage contribution of each compound to the corresponding total pool in each plant-level biological sample. Because relative proportions are compositional and interdependent, compositional data were analyzed after centered log-ratio transformation [54].

Multivariate patterns of metabolite and transcript profiles were explored through principal component analysis (PCA) and hierarchical cluster analysis (HCA) using log2-transformed and autoscaled matrices. HCA was based on Euclidean distances and Ward’s minimum variance clustering method [55]. Whole-profile differences associated with Treatment, Time, and their interaction were explored through permutational multivariate analysis of variance (PERMANOVA) on Euclidean distance matrices [56]. Factorial terms were fitted sequentially in the order of Treatment, Time, and Treatment × Time. Differences between treatment-assigned groups within each sampling time were also explored through time-specific PERMANOVA when appropriate. Because each experimental condition was represented by a single enclosure, experimental condition and enclosure were not independently replicated, preventing their respective contributions from being statistically separated. Accordingly, all linear model and PERMANOVA *p*-values describe patterns observed within this experiment and should not be interpreted as enclosure-independent tests of a causal PPM effect.

To describe the progressive divergence between control and infested profiles during the experiment, Euclidean distances between treatment centroids were calculated at each time point for metabolite and TPS transcript matrices and normalized to the final distance observed at 195 DPI. Pearson correlation coefficients were then calculated between *TPS* transcript abundance and the corresponding putatively associated terpenoid products or diterpene resin acids defined in Table 1 using log2-transformed values. Correlation *p* values were adjusted using the Benjamini–Hochberg procedure when multiple transcript–metabolite relationships were summarized. Because values from both experimental conditions and all sampling times were pooled, these correlations may partly reflect common condition- or time-related trends and cannot establish direct gene–product relationships. Statistical significance was considered at *p* < 0.05 or at *q* < 0.05 after false discovery rate correction, where applicable.

## Figures and Tables

**Figure 1:**
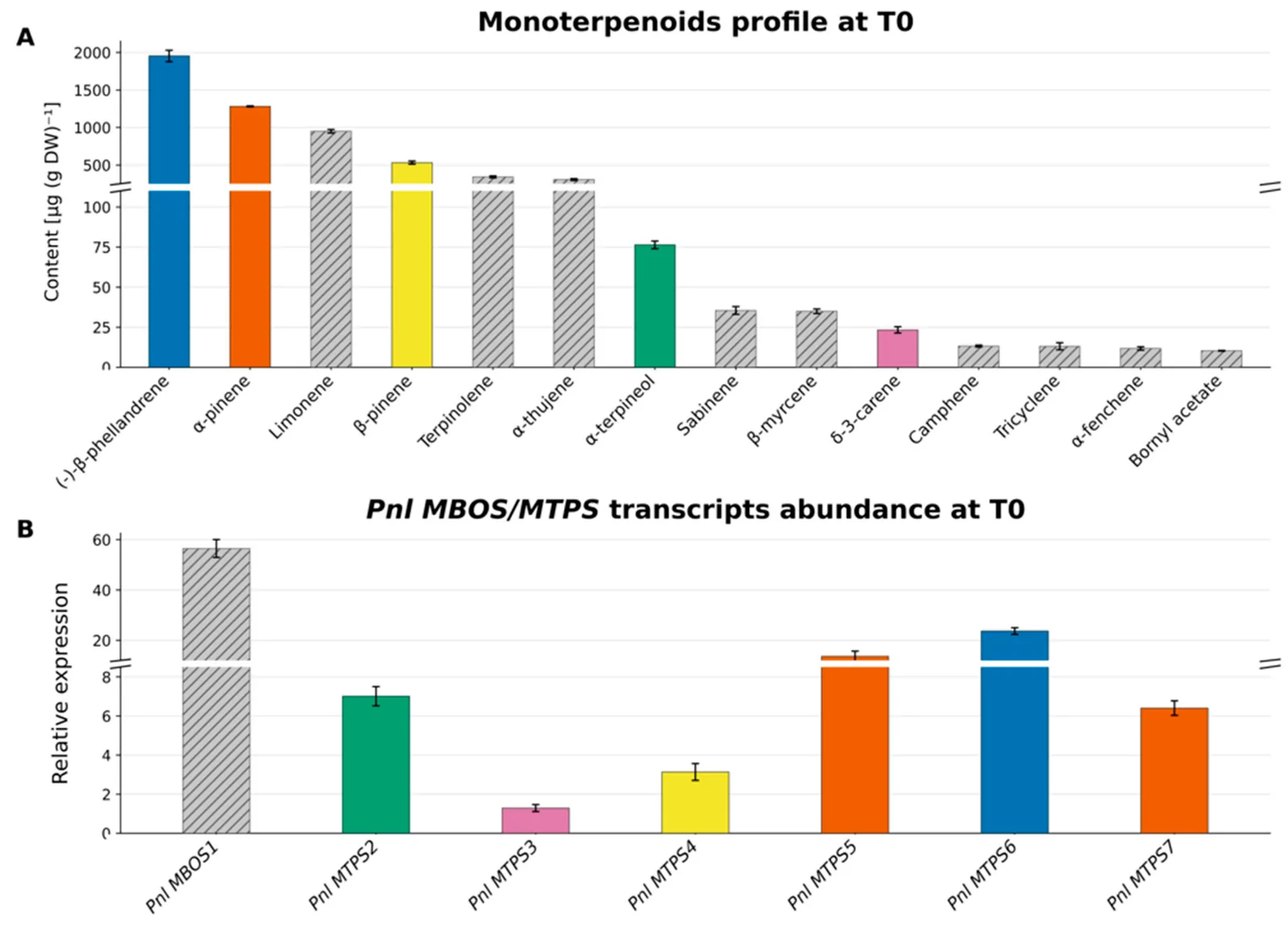
Monoterpenoid profile (**A**) and terpene synthase transcripts abundances (**B**) in needles of non-infested control Calabrian pine saplings at T0, immediately before pine processionary moth (PPM) infestation. DW, needle dry weight; MBOS, 2-methyl-3-buten-2-ol synthase gene; *MTPS*, monoterpene synthase gene. Data are untransformed means ± SEM of three plant-level biological samples from control saplings sampled at T0. Matched colors of the bars in panels A and B indicate the putative MTPS–product associations defined in Table 1. Hatched gray bars indicate genes and/or metabolites with no putative linkage or no quantified counterpart under the reported analytical conditions.

**Figure 2:**
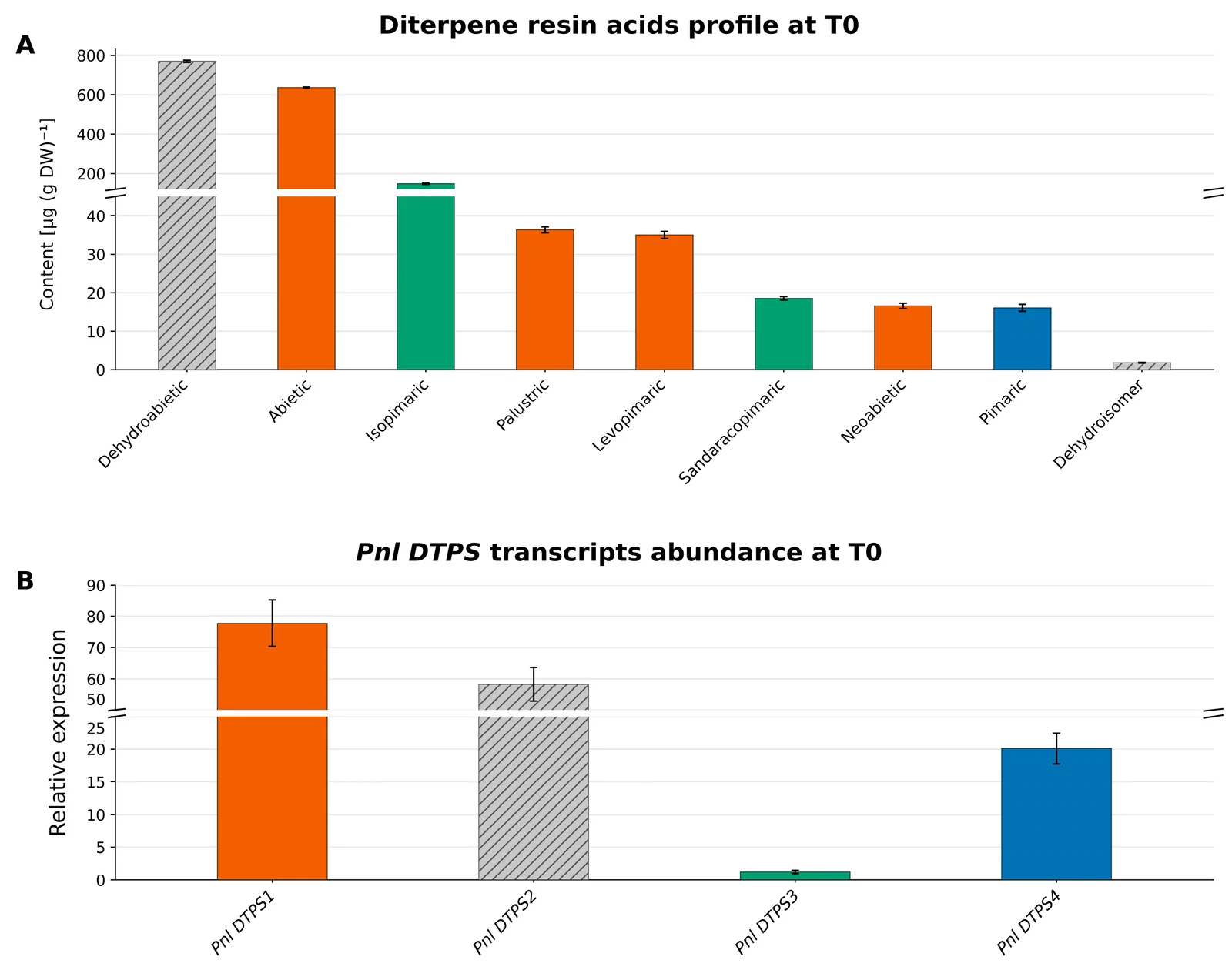
Diterpene resin acid profile (**A**) and diterpene synthase transcript abundance (**B**) in needles of non-infested control Calabrian pine saplings at T0, immediately before pine processionary moth (PPM) infestation. DRA, diterpene resin acid; DW, needle dry weight; *DTPS*, diterpene synthase gene. Data are untransformed means ± SEM of three plant-level biological samples from control saplings sampled at T0. Matched colors of the bars in panels A and B indicate the putative pathway-level DTPS–DRA associations defined in Table 1. Hatched gray bars indicate genes and/or metabolites with no putative linkage or no assigned counterpart under the reported analytical conditions.

**Figure 3:**
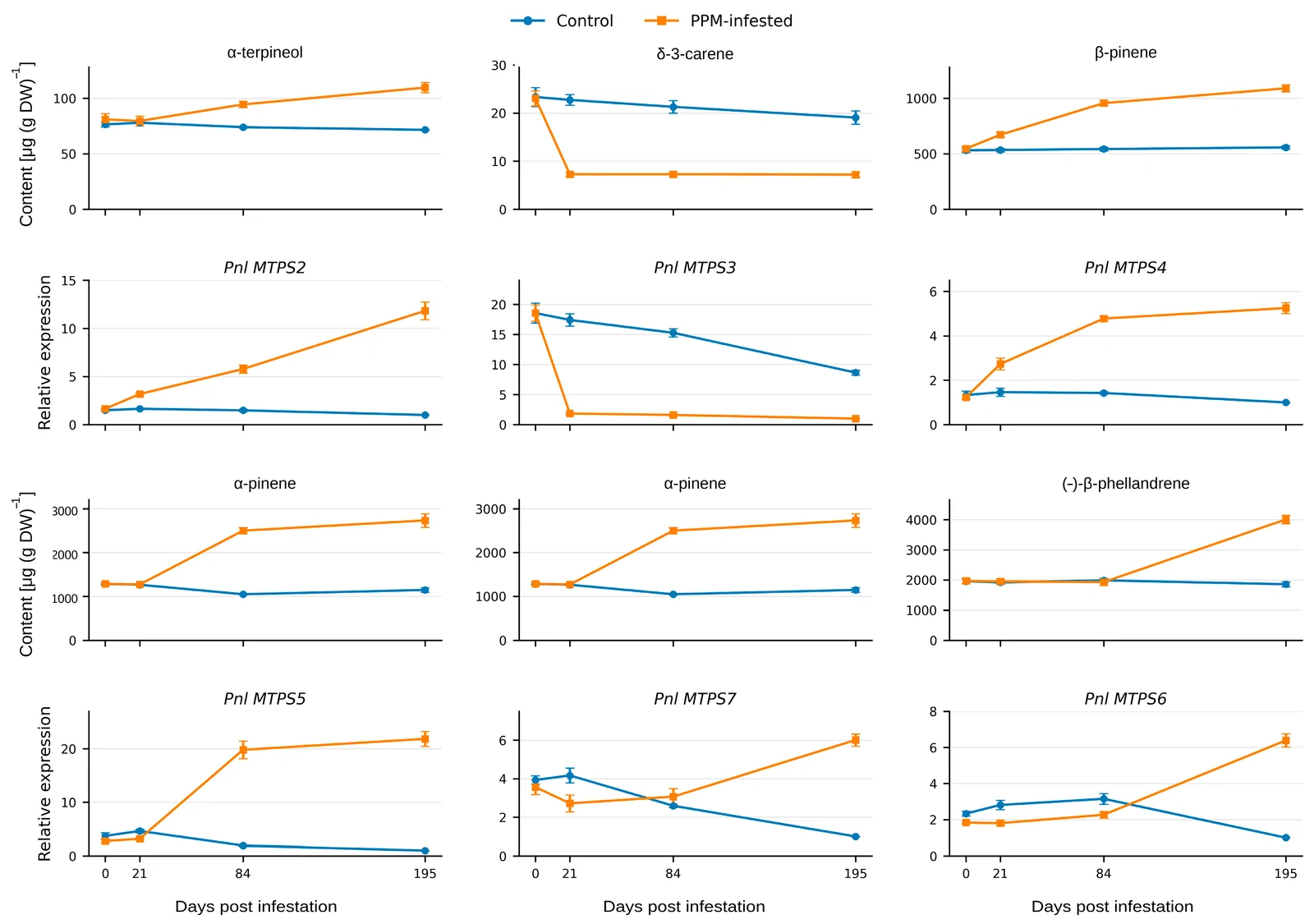
Time-course profiles of monoterpenoids and transcript abundances of their respective putatively associated monoterpene synthase (MTPS) genes in needles of Calabrian pine saplings either exposed to pine processionary moth (PPM) infestation in one enclosure (orange lines) or to pest-free control conditions in a separate enclosure (blue lines). DW, needle dry weight. From top to bottom, each monoterpenoid profile is followed by the profile of its putatively associated transcript according to Table 1. The α-pinene profile is shown twice to separately display its putative associations with *Pnl MTPS5* and *Pnl MTPS7* (Table 1). Data are untransformed means ± SEM of three plant-level biological samples. Statistical results are reported in Table S3 for monoterpenoids and Table S4 for *Pnl MTPS* transcripts.

**Figure 4:**
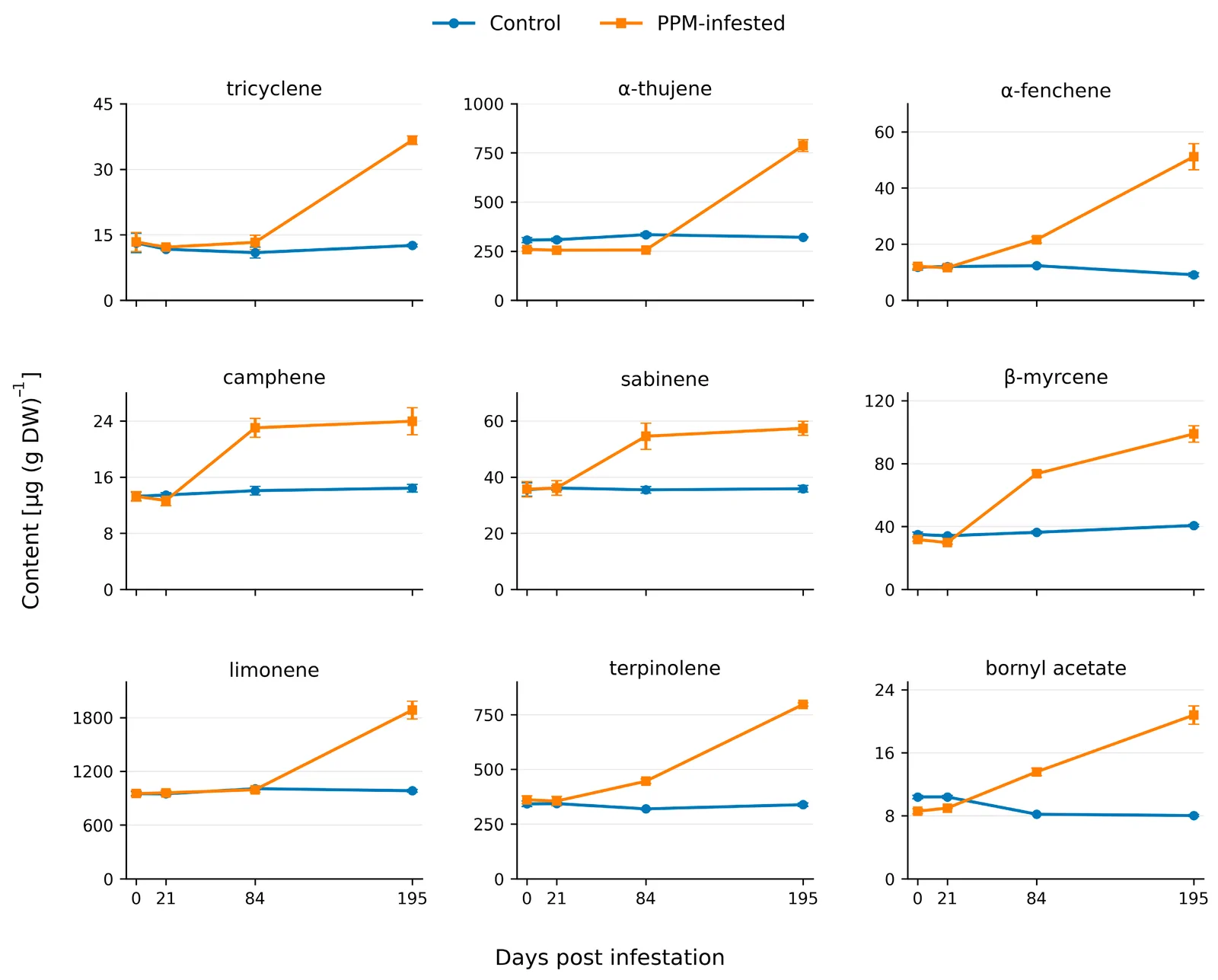
Time-course profiles of monoterpenoids lacking a specific putative association with a monoterpene synthase gene in needles of Calabrian pine saplings either exposed to pine processionary moth (PPM) infestation in one enclosure (orange lines) or to pest-free control conditions in a separate enclosure (blue lines). DW, needle dry weight. Data are untransformed means ± SEM of three plant-level biological samples. Statistical results are reported in Table S3.

**Figure 5:**
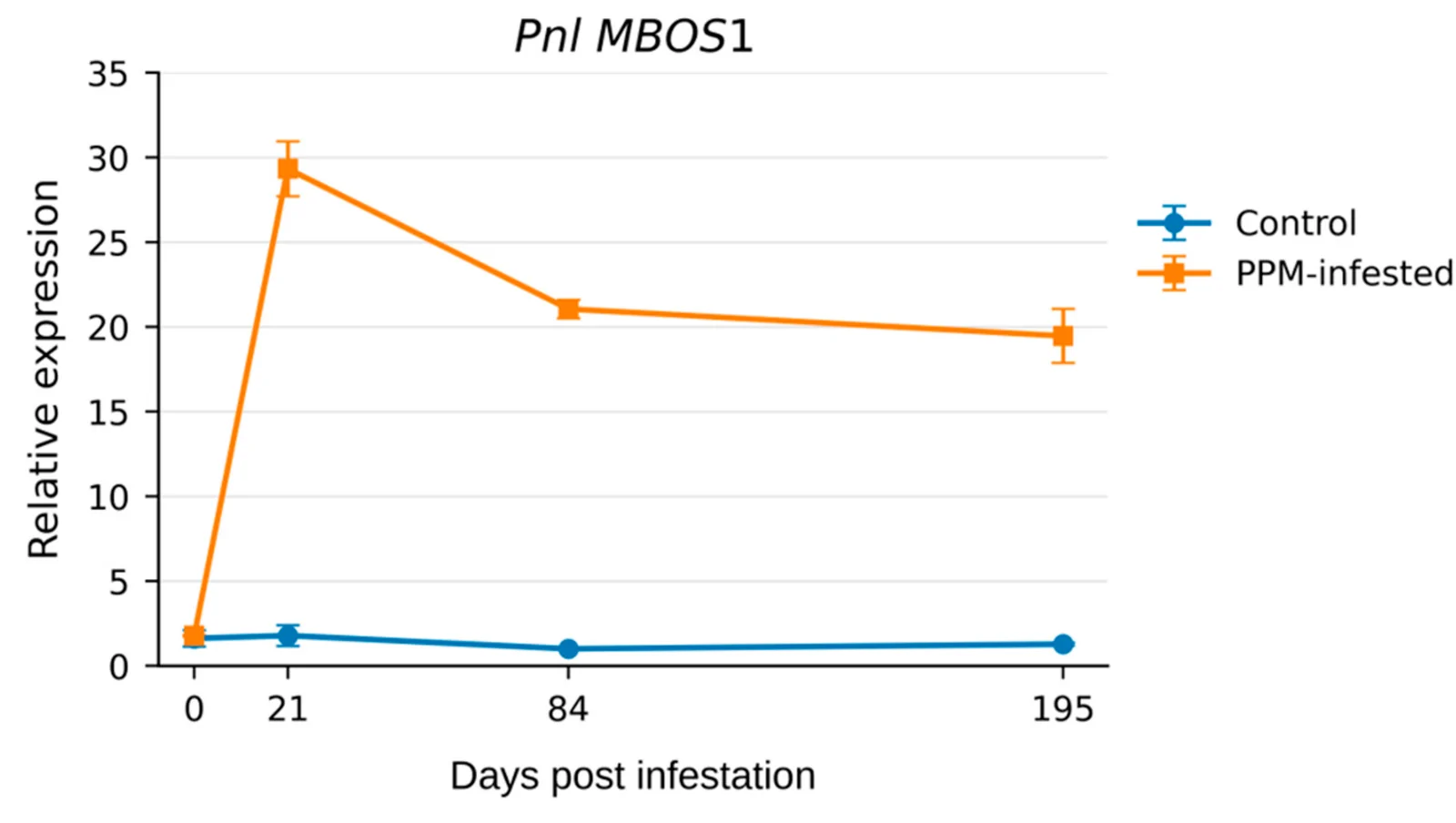
Time-course profile of transcript abundance of 2-methyl-3-buten-2-ol synthase gene 1 (*Pnl MBOS1*) in needles of Calabrian pine saplings either exposed to pine processionary moth (PPM) infestation in one enclosure (orange line) or to pest-free control conditions in a separate enclosure (blue line). Data are untransformed means ± SEM of three plant-level biological samples. Statistical results are reported in Table S4.

**Figure 6:**
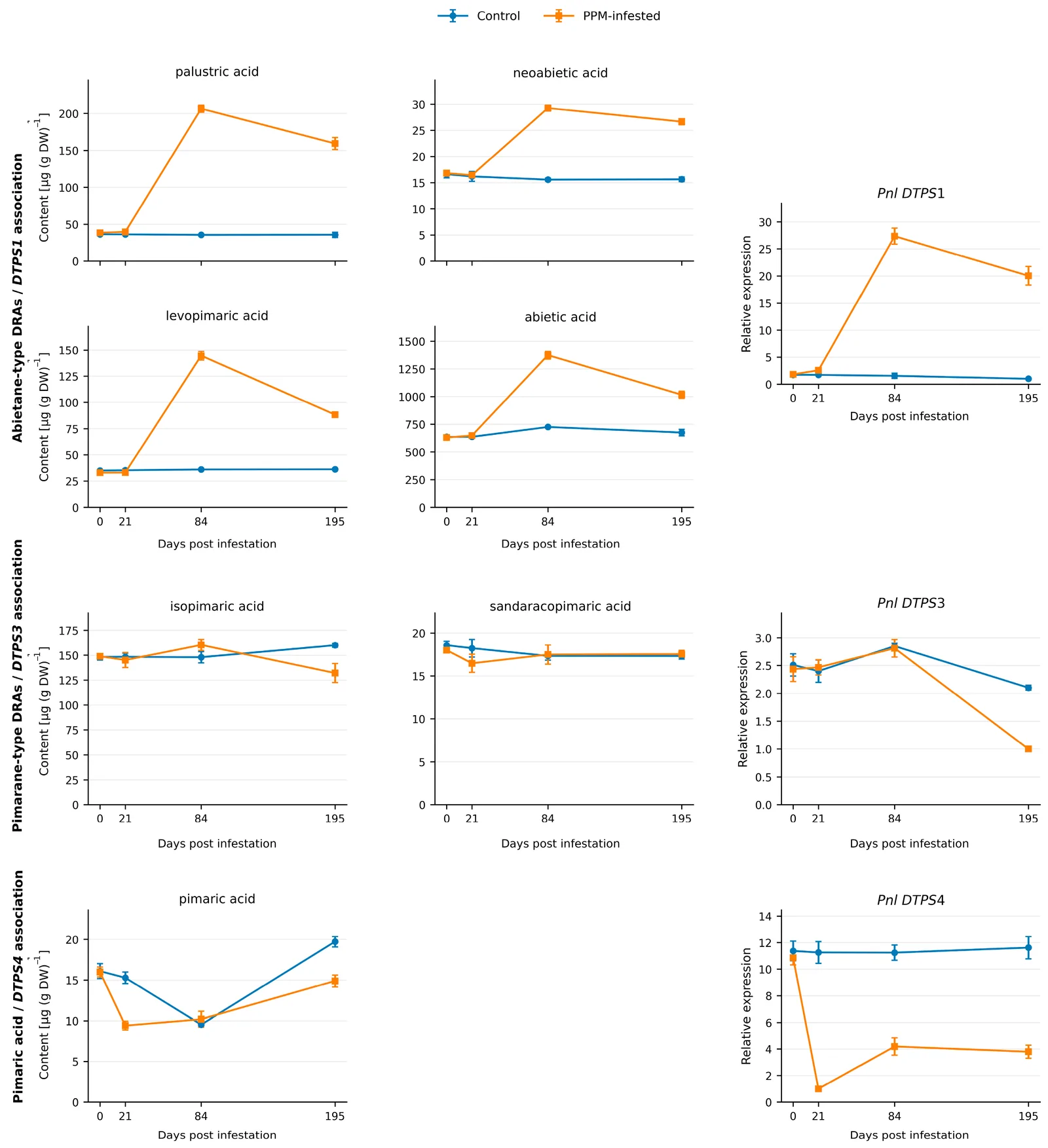
Time-course profiles of diterpene resin acids (DRAs) and transcript abundances of their respective putatively associated diterpene synthase (*DTPS*) genes in needles of Calabrian pine saplings either exposed to pine processionary moth (PPM) infestation in one enclosure (orange lines) or to pest-free control conditions in a separate enclosure (blue lines). DW, needle dry weight. Each set of metabolite panels is grouped with its corresponding transcript panel according to the pathway-level associations defined in Table 1. Data are untransformed means ± SEM of three plant-level biological samples. Statistical results are reported in Table S5 for diterpene resin acids and Table S6 for *Pnl DTPS* transcripts.

**Figure 7:**
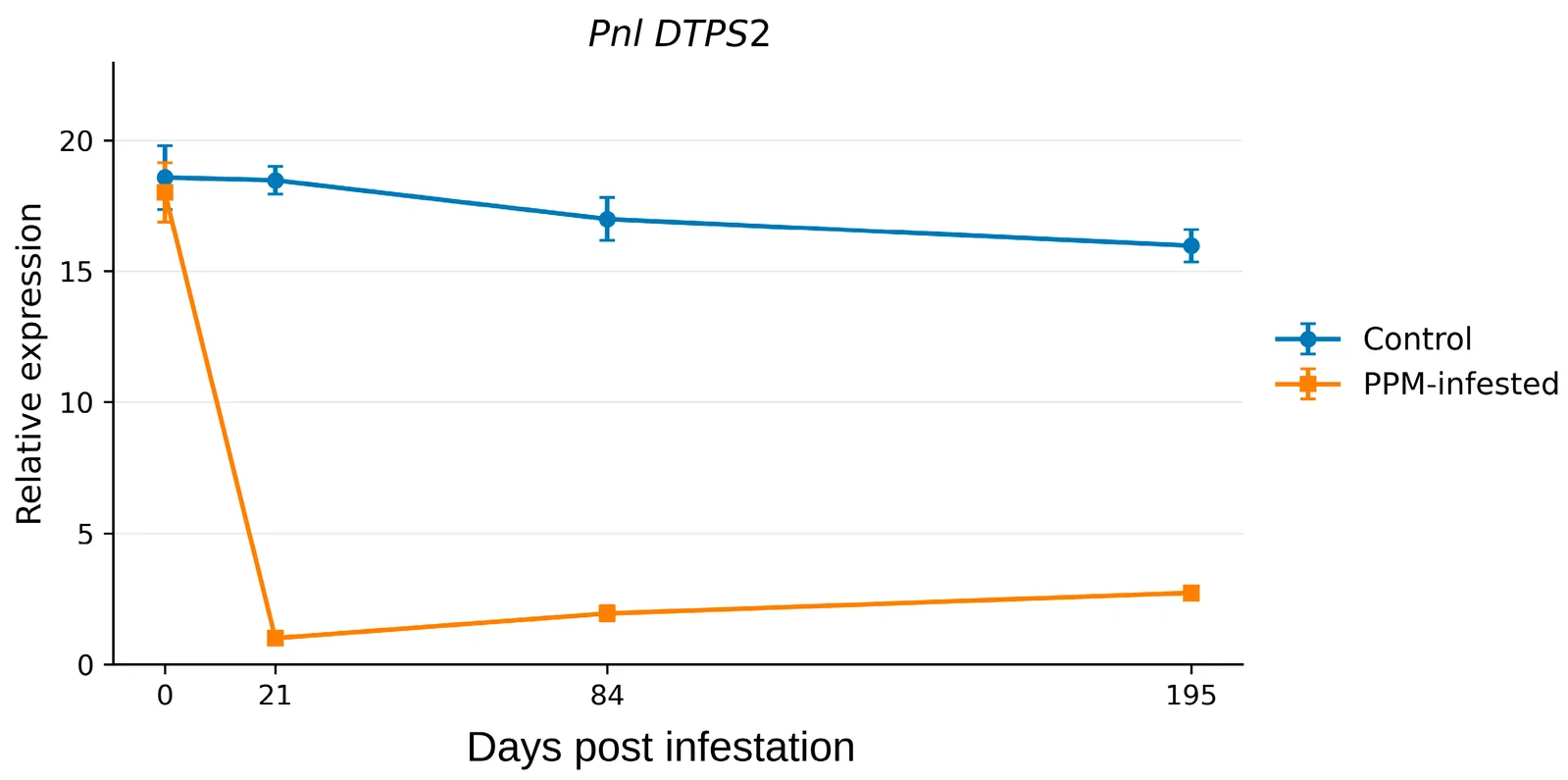
Time-course profile oftranscript abundance of diterpene synthase gene 2 (*Pnl DTPS2*) in needles of Calabrian pine saplings either exposed to pine processionary moth (PPM) infestation in one enclosure (orange line) or to pest-free control conditions in a separate enclosure (blue line). Data are untransformed means ± SEM of three plant-level biological samples. Statistical results are reported in Table S6.

**Figure 8:**
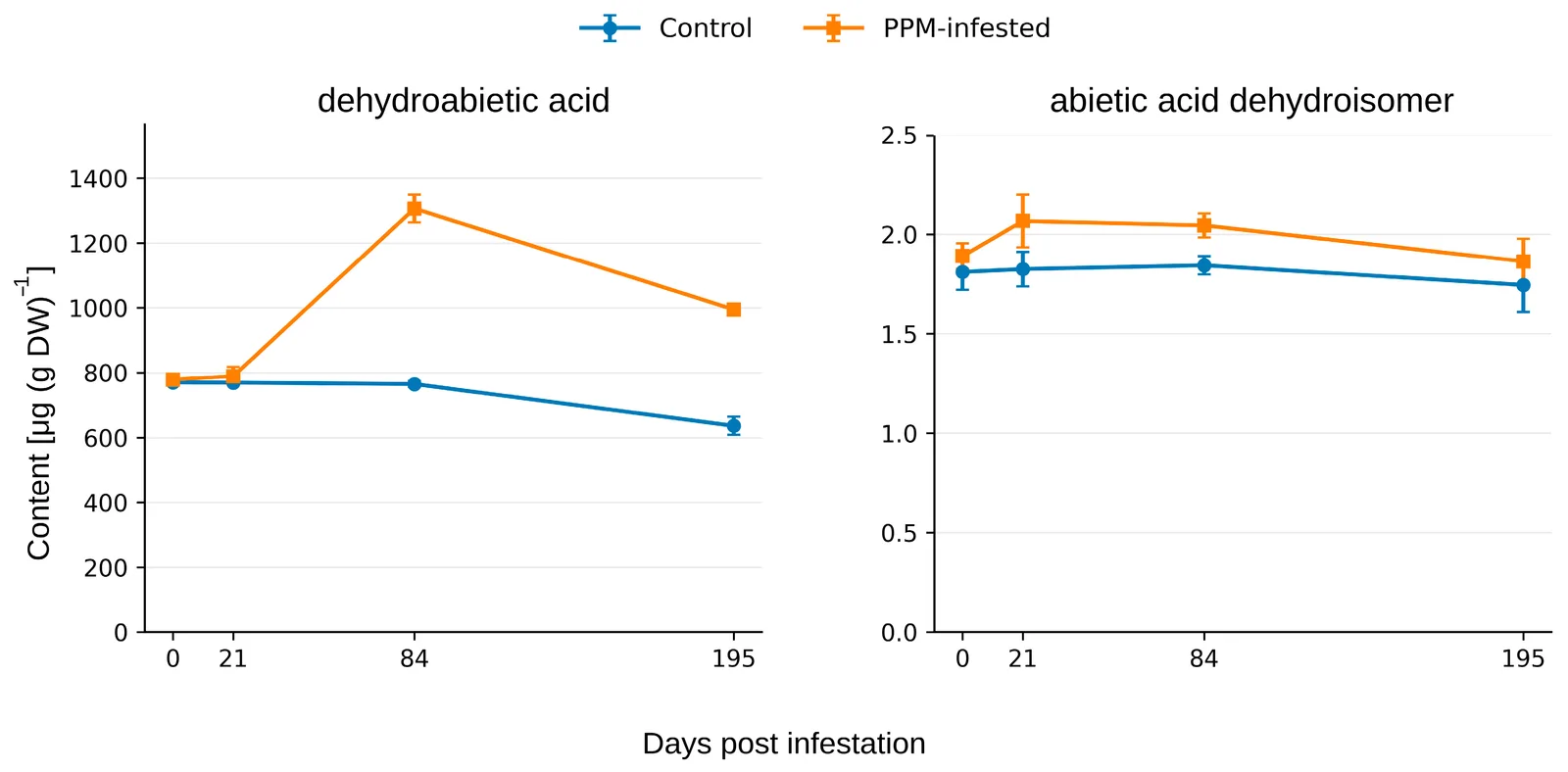
Time-course profiles of diterpene resin acids lacking a specific putative association with a diterpene synthase gene in needles of Calabrian pine saplings either exposed to pine processionary moth (PPM) infestation in one enclosure (orange lines) or to pest-free control conditions in a separate enclosure (blue lines). DW, needle dry weight. Data are untransformed means ± SEM of three plant-level biological samples. Statistical results are reported in Table S5.

**Figure 9:**
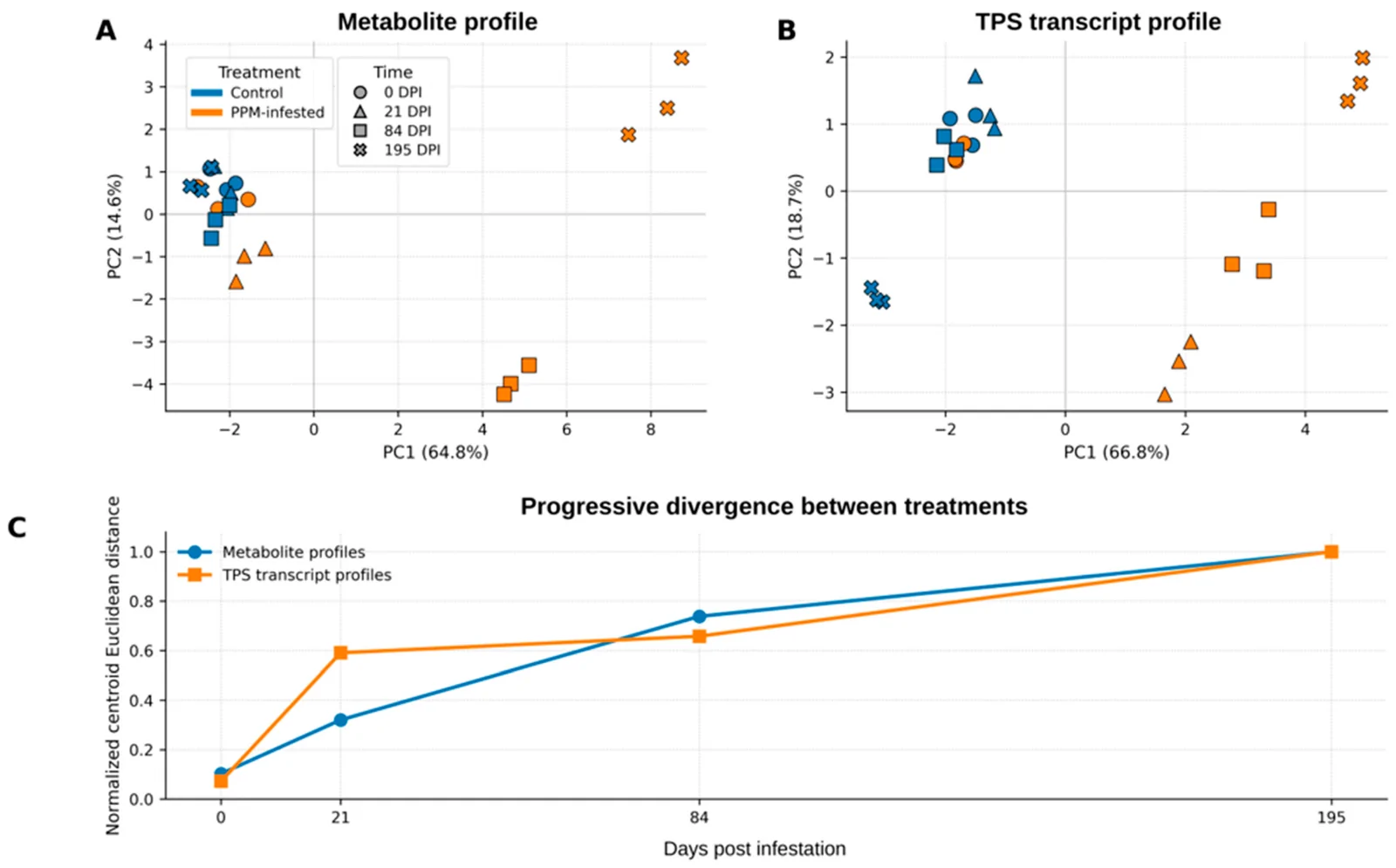
Multivariate overview of metabolic and transcriptional profiles during the pine processionary moth (PPM) infestation time-course in Calabrian pine needles. (**A**) Principal component analysis (PCA) of log2-transformed and autoscaled metabolite profiles, including all quantified monoterpenoids and diterpene resin acids. (**B**) PCA of log2-transformed and autoscaled terpene synthase (TPS) transcript profiles. (**C**) Normalized Euclidean distances between condition centroids over the infestation time-course, calculated separately for metabolite and *TPS* transcripts matrices. Each point in panels (**A**,**B**) represents one plant-level biological sample; distances in panel (**C**) were normalized to the corresponding value at 195 days post-infestation.

**Table 1:** Putative associations between individual terpenoids or terpenoid classes and terpene synthase genes analyzed in Calabrian pine. Associations were inferred from phylogenetic placement, sequence similarity, and functional characterization of homologous terpene synthases from other *Pinus* species.

Terpenoid Product or Class Considered in This Study	Calabrian Pine Gene(s)	Putative Association	Functionally Characterized Gene(s)/Pinus Species	Reference
2-Methyl-3-buten-2-ol (MBO; major product; isoprene as minor product)	*Pnl MBOS1*	Hemiterpene synthase activity inferred from a characterized MBO synthase that converts dimethylallyl diphosphate mainly into MBO.	MBO synthase, *Pinus sabiniana*	[36]
α-Terpineol	*Pnl MTPS2*	Monoterpene synthase putatively associated with α-terpineol formation.	PcTPS-αterp, *P. contorta*; PbTPS-αterp, *P. banksiana*	[37]
δ-3-Carene (3-carene)	*Pnl MTPS3*	Monoterpene synthase putatively associated with 3-carene formation.	PcTPS(+)3car1, *P. contorta*; PbTPS(+)3car1/PbTPS(+)3car2, *P. banksiana*	[37]
β-Pinene	*Pnl MTPS4*	Monoterpene synthase putatively associated with β-pinene formation.	PcTPS(−)βpin1/PcTPS-mono1, *P. contorta*; PbTPS(−)βpin1/PbTPS(−)βpin2, *P. banksiana*	[37]
α-Pinene	*Pnl MTPS5*; *Pnl MTPS7*	Both Calabrian pine genes were putatively associated with α-pinene formation.	PcTPS(+)αpin1/PcTPS(−)αpin1, *P. contorta*; PbTPS(+)αpin1/PbTPS(−)αpin1/PbTPS(−)α/βpin1, *P. banksiana*	[37]
(−)-β-Phellandrene	*Pnl MTPS6*	Monoterpene synthase putatively associated with β-phellandrene formation.	PcTPS(−)βphell1/PcTPS(−)βphell2, *P. contorta*; PbTPS(−)βphell1, *P. banksiana*	[37]
Abietane-type DRAs (abietic acid, levopimaric acid, palustric acid, and neoabietic acid)	*Pnl DTPS1*	Pathway-level association with abietane-type DRA biosynthesis through the corresponding diterpene olefin precursors: abietadiene, levopimaradiene, palustradiene, and neoabietadiene.	PcLAS1/PcLAS2, *P. contorta*; PbLAS1, *P. banksiana*	[38]
No DRA assigned; product relationship unresolved	*Pnl DTPS2*	No terpenoid product was assigned because the closest functionally tested class-I diTPS candidates did not yield obvious diterpene products in coupled assays.	PbmdiTPS1, *P. banksiana*; PcmdiTPS1/PcmdiTPS2/PcmdiTPS3, *P. contorta*	[38]
Pimarane-type DRAs (isopimaric acid and sandaracopimaric acid)	*Pnl DTPS3*	Pathway-level association through the corresponding olefin precursors isopimaradiene and sandaracopimaradiene.	PcmISO1, *P. contorta*; PbmISO1, *P. banksiana*	[38]
Pimarane-type DRA (pimaric acid)	*Pnl DTPS4*	Pathway-level association through the corresponding olefin precursor pimaradiene.	PcmPIM1, *P. contorta*; PbmPIM1, *P. banksiana*	[38]

Note: DRA, diterpene resin acid; MBO, 2-methyl-3-buten-2-ol; TPS, terpene synthase. For diterpenoids, associations indicate pathway-level links between *Pnl DTPS* genes and diterpene resin acids derived from the corresponding olefin precursors rather than direct DTPS products.

**Table 2:** Permutational multivariate analysis of variance (PERMANOVA) summary of Treatment, Time, and Treatment × Time differences on metabolites and terpene synthase (*TPS*) transcript profiles. df, degrees of freedom; SS, sum of squares; R^2^, effect size; the *p* values were obtained from 9999 permutations.

Dataset	Effect	df	Residual df	SS	R^2^	Pseudo-F	*p*
Metabolite profiles	Treatment	1	16	138.963	0.252	32.913	<0.001
Metabolite profiles	Time	3	16	169.906	0.308	13.414	<0.001
Metabolite profiles	Treatment × Time	3	16	175.576	0.318	13.862	<0.001
TPS transcript profiles	Treatment	1	16	101.543	0.385	211.600	<0.001
TPS transcript profiles	Time	3	16	54.947	0.208	38.168	<0.001
TPS transcript profiles	Treatment × Time	3	16	99.832	0.378	69.345	<0.001

## Data Availability

The data supporting the findings of this study are available within the article and its Supplementary Material.

## Supplementary Materials

The following supporting information can be downloaded at https://www.mdpi.com/article/10.3390/ijms27219775/s1.
