# Supplementary material for: Terpenoid Accumulation and Terpene Synthase Gene Expression Are Coordinately Modulated in Calabrian Pine (Pinus nigra subsp. laricio) Following Infestation by the Pine Processionary Moth (Thaumetopoea pityocampa)
Source: Int J Mol Sci. 2026 Oct 30;27(21):9775. doi: 10.3390/ijms27219775 (PMC13762599; doi:10.3390/ijms27219775)
Supplement: Supplementary file 1 [file ijms-27-09775-s001.zip › ijms-4589942-supplementary.pdf]

Supplementary Materials

Supplementary Figures

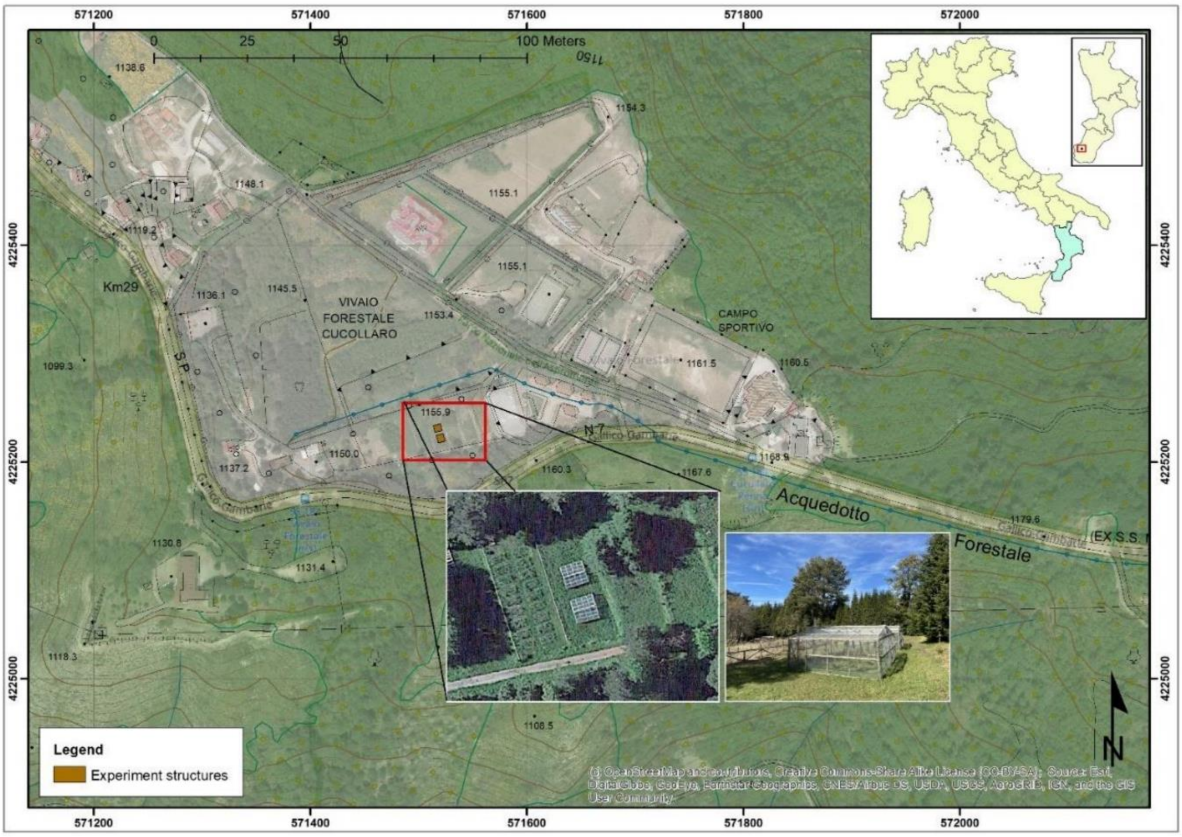

Figure S1. Experimental site of the semi-natural pine processionary moth infestation trial. Location of the Calabria Regional Observatory for Biodiversity, Santo Stefano in Aspromonte, Cucullaro locality, within the Aspromonte National Park, Southern Italy. The red box indicates the area containing the experimental greenhouse-type enclosures, shown in the inset images.

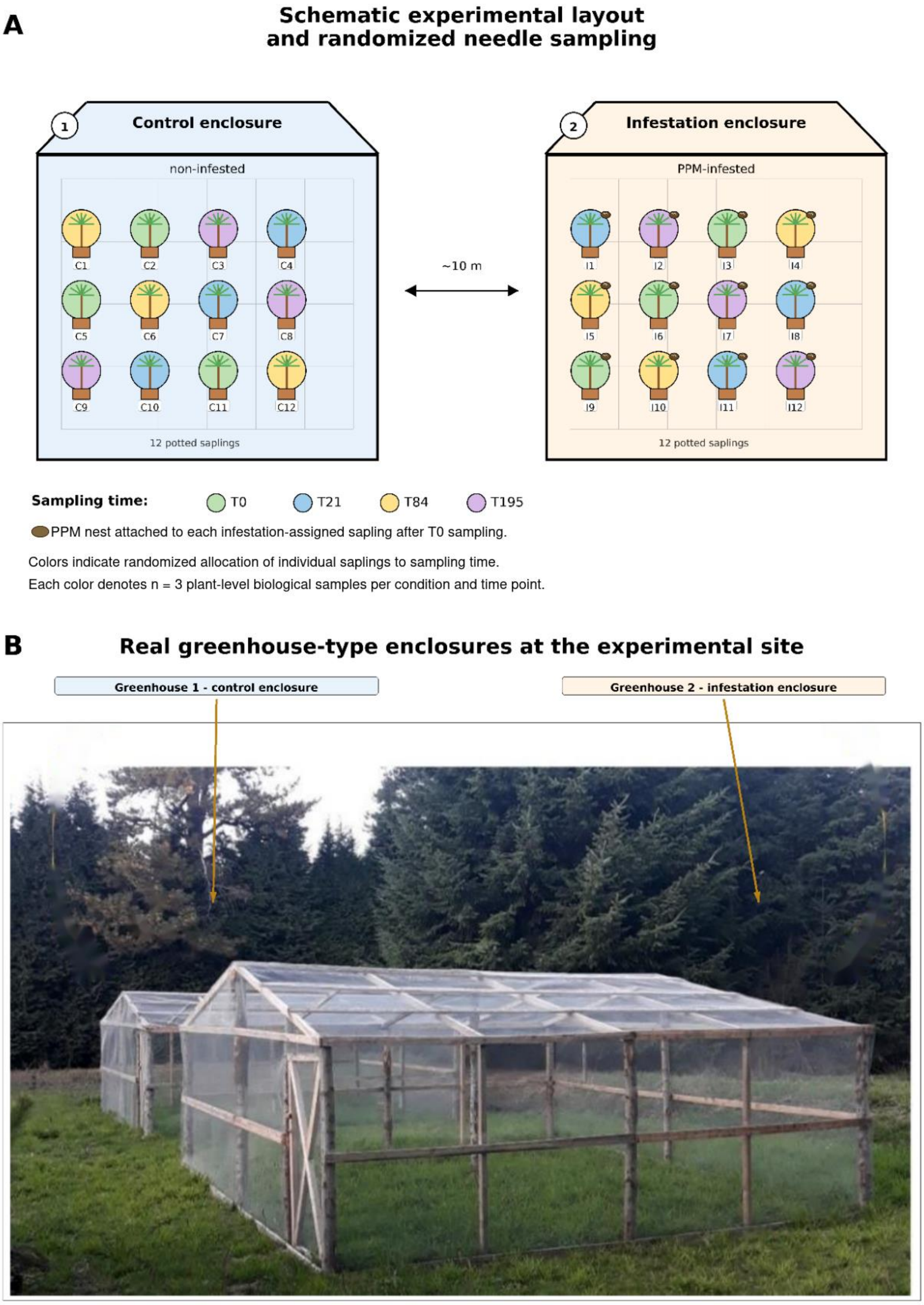

Figure S2. Experimental layout of the semi-natural pine processionary moth (PPM) infestation trial. (A) Schematic representation of the two greenhouse-type enclosures used for non-infested control and PPM-infested Calabrian pine saplings. Each enclosure contained 12 potted saplings, randomly allocated to four sampling times (n = 3 plant-level biological samples per condition and time point). (B) Real greenhouse-type enclosures at the experimental site.

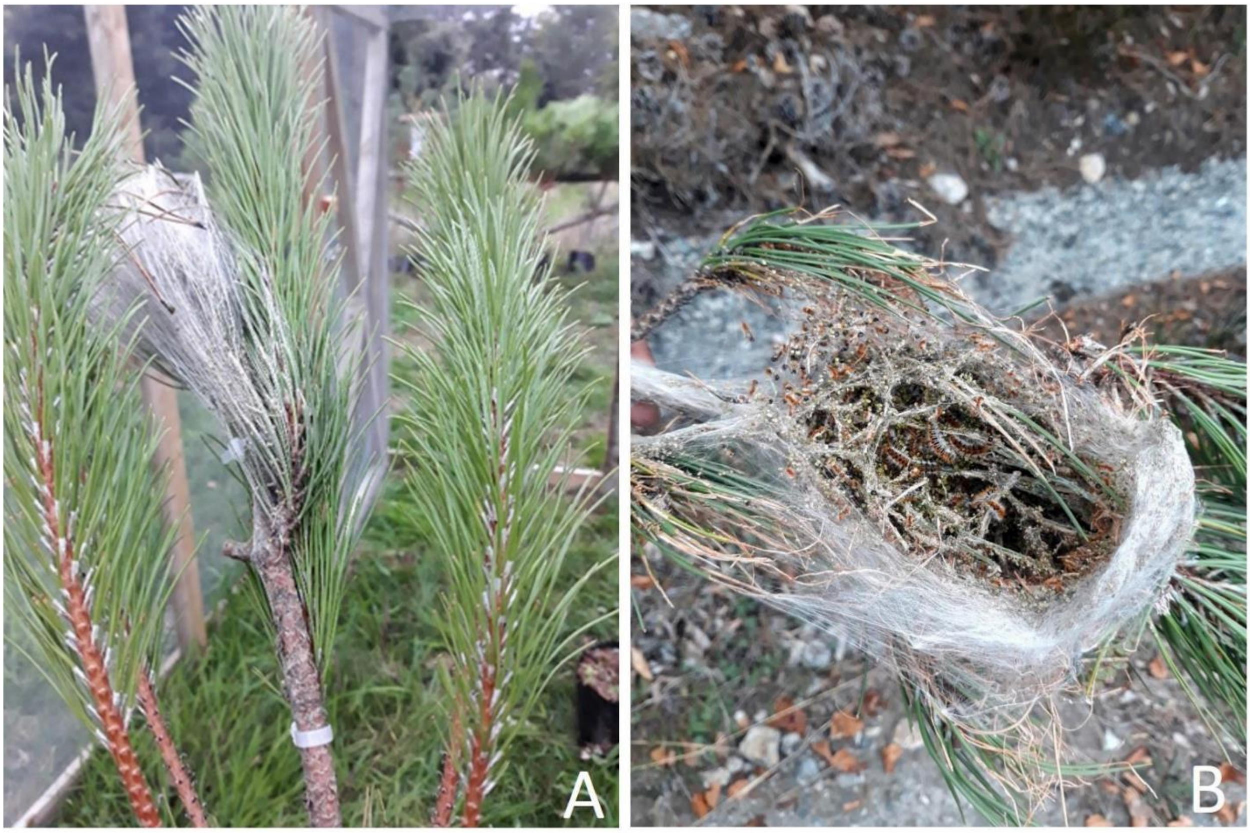

Figure S3. Pine processionary moth (PPM) nest and larvae used for experimental infestation. (A) PPM nest attached to a Calabrian pine sapling with plastic ties. (B) Larvae observed inside a sampled nest at the start of the experimental infestation.

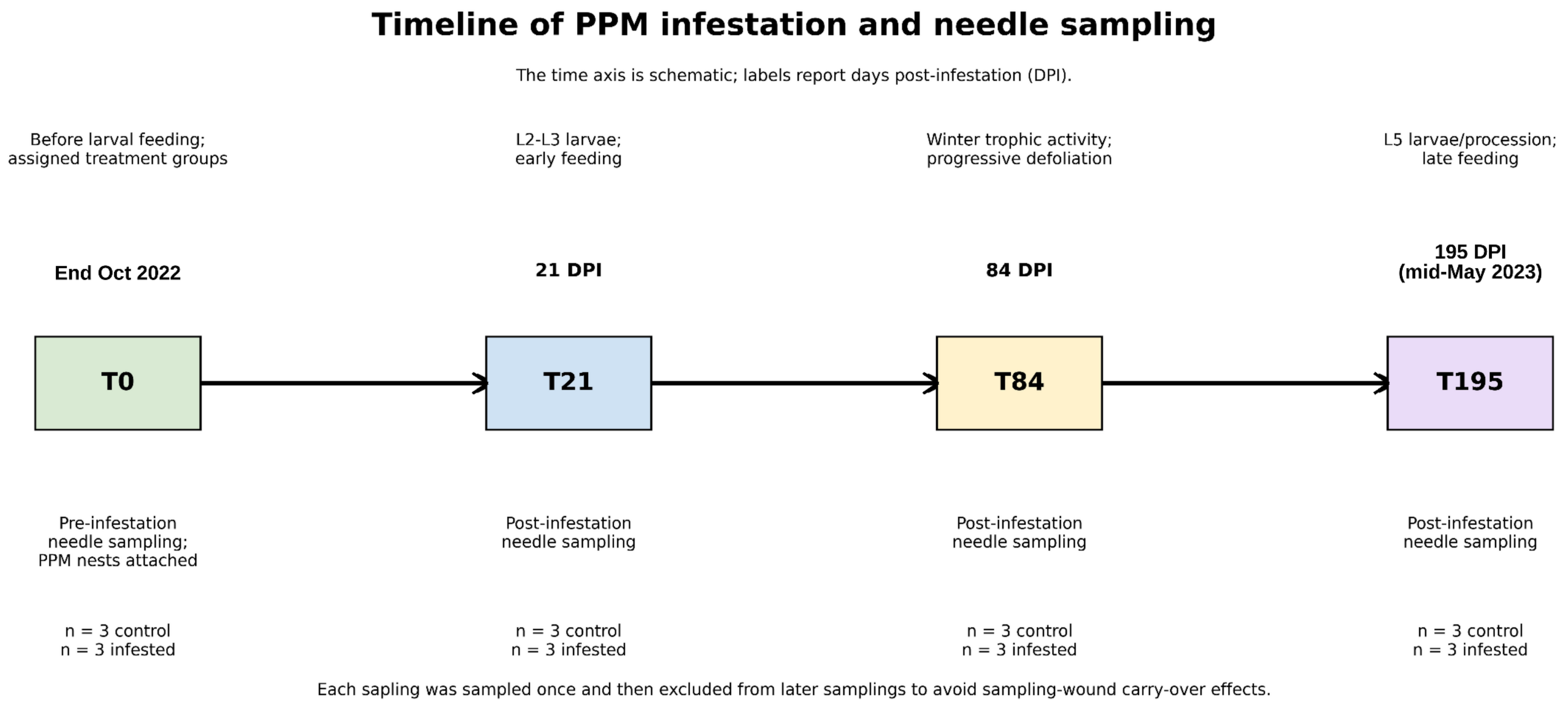

Figure S4. Timeline of pine processionary moth (PPM) infestation and pine needle sampling. T0 indicates pre-infestation sampling at the end of October 2022, immediately before PPM nests were attached to infestation-assigned saplings. T21, T84 and T195 indicate 21, 84 and 195 days post-infestation, respectively. The sampling times were selected to represent the broad developmental and feeding phases shown in the timeline: early feeding by L2–L3 larvae at T21, winter trophic activity with progressive defoliation at T84, and late feeding by L5 larvae and the preceding procession phase at T195. At each time point, three control-assigned and three infestation-assigned saplings were sampled as distinct plant-level biological units. Each sapling was sampled once and then excluded from subsequent sampling to avoid carry-over effects from sampling-wound. DPI, days post-infestation.

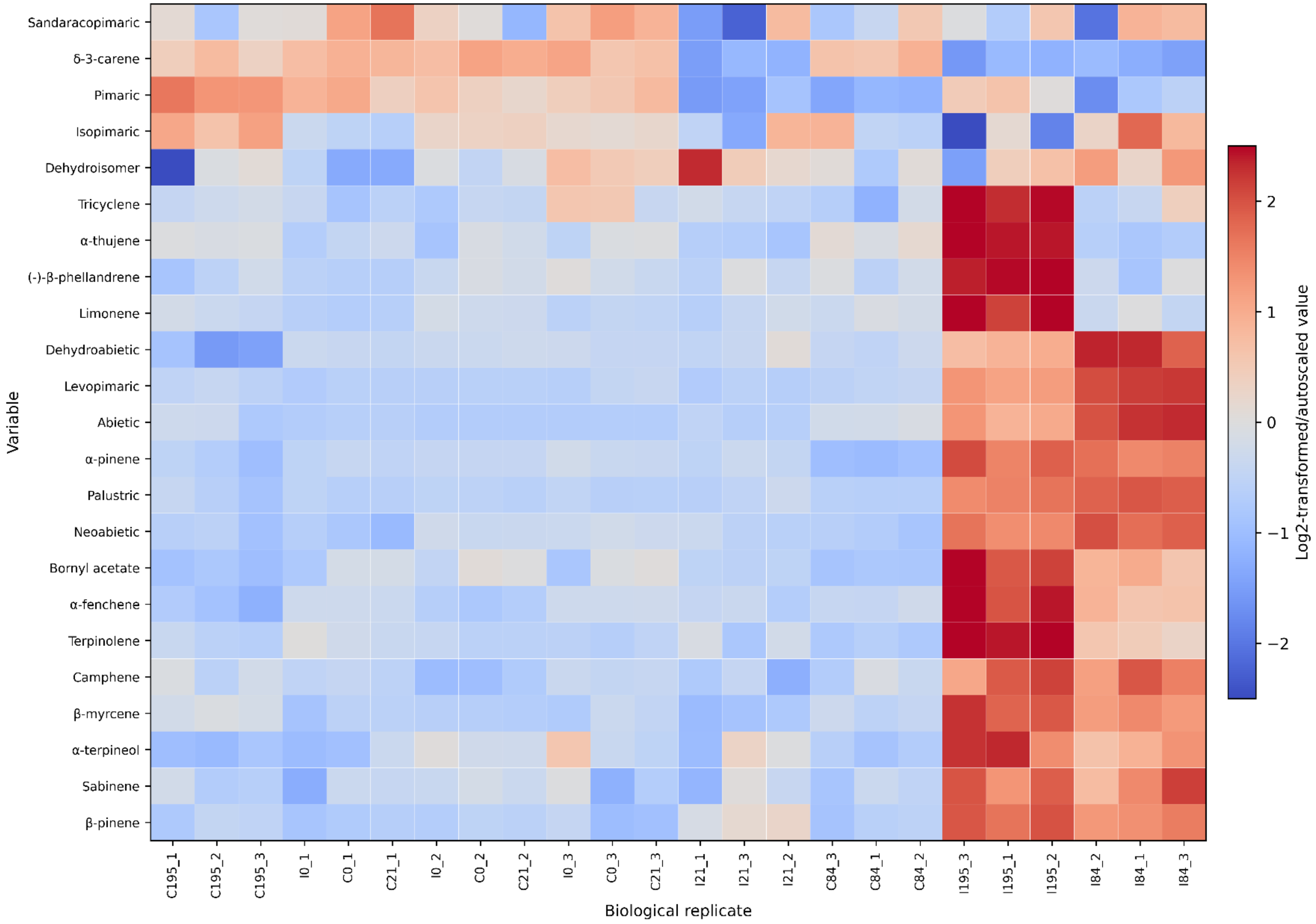

Figure S5. Hierarchical clustering heatmap of metabolite profiles across the time course of pine processionary moth infestation. The heatmap was generated from the complete metabolite matrix, including all quantified monoterpenoids and diterpene resin acids, after log2 transformation and autoscaling. Clustering was based on Euclidean distances and Ward's minimum-variance method. Columns represent plant-level biological samples and rows represent metabolites; the colours indicates log2-transformed and autoscaled values. C, control-assigned saplings; I, infestation-assigned saplings.

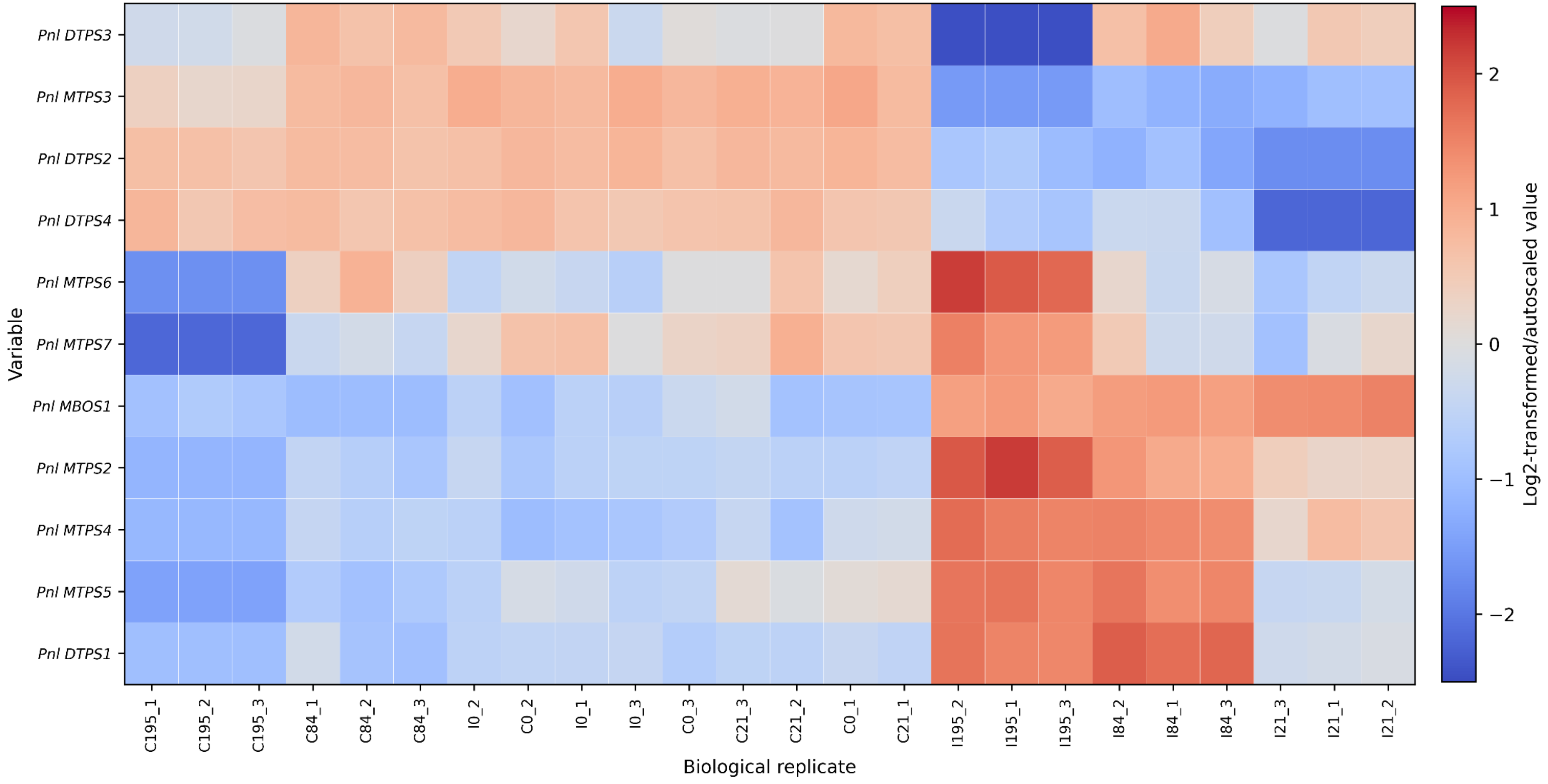

Figure S6. Hierarchical clustering heatmap of terpene synthase (TPS) transcript profiles across the time course of pine processionary moth infestation. The heatmap was generated from the complete TPS transcript matrix, including *Pnl MBOS1*, *Pnl MTPS2*–*Pnl MTPS7* and *Pnl DTPS1*–*Pnl DTPS4*, after log2 transformation and autoscaling. Clustering was based on Euclidean distances and Ward's minimum-variance method. Columns represent plant-level biological samples and rows represent transcripts; the color scale indicates log2-transformed and autoscaled values. C, control-assigned saplings; I, infestation-assigned saplings.

Supplementary Tables

Table S1. Monoterpenoid concentrations [μg (g dry weight)$^{-1}$] and monoterpene synthase (*MTPS*) transcript abundances (relative expression) in needles of non-infested control Calabrian pine saplings immediately before pine processionary moth infestation.

Variable	n	Mean ± SEM (untransformed)	Relative contribution (% of pool)	Mean ± SEM (log2-transformed)
(-)-β-phellandrene	3	1954.3 ± 75.66	35.0	10.93 ± 0.057
α-pinene	3	1281.2 ± 6.083	22.9	10.32 ± 0.007
limonene	3	950.5 ± 24.04	17.0	9.892 ± 0.037
β-pinene	3	531.5 ± 20.39	9.5	9.052 ± 0.056
terpinolene	3	342.1 ± 12.79	6.1	8.416 ± 0.053
α-thujene	3	306.7 ± 12.30	5.5	8.258 ± 0.059
α-terpineol	3	76.41 ± 2.357	1.4	6.254 ± 0.045
sabinene	3	35.52 ± 2.394	0.6	5.144 ± 0.101
β-myrcene	3	34.97 ± 1.433	0.6	5.126 ± 0.058
δ-3-carene	3	23.34 ± 1.971	0.4	4.534 ± 0.125
camphene	3	13.26 ± 0.611	0.2	3.725 ± 0.068
tricyclene	3	13.15 ± 2.205	0.2	3.678 ± 0.234
α-fenchene	3	11.82 ± 1.028	0.2	3.552 ± 0.132
bornyl acetate	3	10.37 ± 0.215	0.2	3.373 ± 0.030
Pnl MBOS1	3	56.45 ± 3.550	—	5.813 ± 0.090
Pnl MTPS2	3	7.010 ± 0.488	—	2.802 ± 0.104
Pnl MTPS3	3	1.289 ± 0.178	—	0.339 ± 0.199
Pnl MTPS4	3	3.137 ± 0.432	—	1.623 ± 0.195
Pnl MTPS5	3	13.66 ± 2.066	—	3.738 ± 0.226
Pnl MTPS6	3	23.71 ± 1.309	—	4.563 ± 0.080
Pnl MTPS7	3	6.404 ± 0.369	—	2.674 ± 0.086

Note: Values (means ± SEM) refer to three plant-level biological samples collected from non-infested control saplings at T0. Relative contribution was calculated only for metabolite rows as the percentage of the total monoterpenoid pool at T0. Log2-transformed values document the scale used for statistical inference, whereas corresponding figures show untransformed means. SEM, standard error of the mean.

Table S2. Diterpene resin acid concentrations [μg (g dry weight)$^{-1}$] and diterpene synthase (DTPS) transcript abundances (relative expression) in needles of non-infested control Calabrian pine saplings immediately before pine processionary moth infestation.

Variable	n	Mean ± SEM (untransformed)	Relative contribution (% of pool)	Mean ± SEM (log2-transformed)
dehydroabietic acid	3	770.1 ± 5.122	45.9	9.589 ± 0.010
abietic acid	3	635.7 ± 2.509	37.9	9.312 ± 0.006
isopimaric acid	3	148.2 ± 2.961	8.8	7.211 ± 0.029
palustric acid	3	36.33 ± 0.777	2.2	5.182 ± 0.031
levopimaric acid	3	34.99 ± 0.921	2.1	5.128 ± 0.038
sandaracopimaric acid	3	18.57 ± 0.466	1.1	4.214 ± 0.037
neoabietic acid	3	16.60 ± 0.697	1.0	4.051 ± 0.062
pimaric acid	3	16.09 ± 0.924	1.0	4.004 ± 0.081
abietic acid dehydroisomer	3	1.810 ± 0.090	0.1	0.852 ± 0.071
Pnl DTPS1	3	77.78 ± 7.429	—	6.268 ± 0.142
Pnl DTPS2	3	58.25 ± 5.406	—	5.851 ± 0.141
Pnl DTPS3	3	1.236 ± 0.231	—	0.259 ± 0.253
Pnl DTPS4	3	20.12 ± 2.341	—	4.311 ± 0.164

Note: Values (means ± SEM) refer to three plant-level biological samples collected from non-infested control saplings at T0. Relative contribution was calculated only for metabolite rows as the percentage of the total diterpene resin acid pool at T0. Log2-transformed values document the scale used for statistical inference, whereas corresponding figures show untransformed means. SEM, standard error of the mean.

Table S3. Linear-model analysis and time-specific treatment contrasts for monoterpenoid concentrations [μg (g dry weight)$^{-1}$] in Calabrian pine needles during pine processionary moth (PPM) infestation. DPI, days post-infestation. *MTPS*, monoterpene synthase gene.

Variable	Assignment	Treatment p	Time p	Treatment × Time p	0 DPI	21 DPI	84 DPI	195 DPI
α-terpineol	Putatively associated with *Pnl MTPS2*	0.332	0.436	<0.001	ns (q=0.443)	ns (q=0.757)	↑*** (q<0.001)	↑*** (q<0.001)
δ-3-carene	Putatively associated with *Pnl MTPS3*	0.928	0.213	<0.001	ns (q=0.928)	↓*** (q<0.001)	↓*** (q<0.001)	↓*** (q<0.001)
β-pinene	Putatively associated with *Pnl MTPS4*	0.553	0.702	<0.001	ns (q=0.553)	↑*** (q<0.001)	↑*** (q<0.001)	↑*** (q<0.001)
α-pinene	Putatively associated with *Pnl MTPS5*/*Pnl MTPS7*	0.951	<0.001	<0.001	ns (q=0.951)	ns (q=0.951)	↑*** (q<0.001)	↑*** (q<0.001)
(-)-β-phellandrene	Putatively associated with *Pnl MTPS6*	0.934	0.715	<0.001	ns (q=0.934)	ns (q=0.934)	ns (q=0.934)	↑*** (q<0.001)
tricyclene	No specific *Pnl MTPS* assignment	0.906	0.628	<0.001	ns (q=0.906)	ns (q=0.906)	ns (q=0.386)	↑*** (q<0.001)
α-thujene	No specific *Pnl MTPS* assignment	<0.001	0.171	<0.001	↓*** (q<0.001)	↓*** (q<0.001)	↓*** (q<0.001)	↑*** (q<0.001)
α-fenchene	No specific *Pnl MTPS* assignment	0.783	0.021	<0.001	ns (q=0.783)	ns (q=0.783)	↑*** (q<0.001)	↑*** (q<0.001)
camphene	No specific *Pnl MTPS* assignment	1.000	0.659	<0.001	ns (q=1.000)	ns (q=0.558)	↑*** (q<0.001)	↑*** (q<0.001)
sabinene	No specific *Pnl MTPS* assignment	0.965	0.994	<0.001	ns (q=0.965)	ns (q=0.965)	↑*** (q<0.001)	↑*** (q<0.001)
β-myrcene	No specific *Pnl MTPS* assignment	0.059	0.009	<0.001	ns (q=0.059)	↓* (q=0.015)	↑*** (q<0.001)	↑*** (q<0.001)
limonene	No specific *Pnl MTPS* assignment	0.990	0.456	<0.001	ns (q=0.990)	ns (q=0.990)	ns (q=0.990)	↑*** (q<0.001)
terpinolene	No specific *Pnl MTPS* assignment	0.303	0.424	<0.001	ns (q=0.404)	ns (q=0.546)	↑*** (q<0.001)	↑*** (q<0.001)
bornyl acetate	No specific *Pnl MTPS* assignment	<0.001	<0.001	<0.001	↓*** (q<0.001)	↓** (q=0.003)	↑*** (q<0.001)	↑*** (q<0.001)

Note: Linear models were fitted separately for each monoterpenoid using log2-transformed concentrations as the response variable and Treatment, Time and Treatment × Time as fixed effects. Reported *p* values refer to Type III ANOVA tests. Time-specific contrasts compare PPM-infested plants with non-infested controls at the same sampling time. *q* values were obtained by Benjamini–Hochberg correction across the four contrasts within each monoterpenoid. Arrows indicate higher (↑) or lower (↓) values in PPM-infested plants relative to controls; ns, not significant. Significance codes are based on *q* values: * $q < 0.05$; ** $q < 0.01$; *** $q < 0.001$. Values plotted in Figures 3 and 4 are untransformed means ± standard error of the mean.

Table S4. Linear-model analysis and time-specific treatment contrasts for 2-methyl-3-buten-2-ol/monoterpene synthase (*MBOS/MTPS*) transcripts abundances (relative expression) in Calabrian pine needles during pine processionary moth (PPM) infestation. DPI, days post-infestation.

Variable	Assignment	Treatment p	Time p	Treatment × Time p	0 DPI	21 DPI	84 DPI	195 DPI
Pnl MBOS1	MBO synthase activity; MBO not quantified	0.447	0.186	<0.001	ns (q=0.447)	↑*** (q<0.001)	↑*** (q<0.001)	↑*** (q<0.001)
Pnl MTPS2	α-terpineol	0.242	<0.001	<0.001	ns (q=0.242)	↑*** (q<0.001)	↑*** (q<0.001)	↑*** (q<0.001)
Pnl MTPS3	δ-3-carene	0.986	<0.001	<0.001	ns (q=0.986)	↓*** (q<0.001)	↓*** (q<0.001)	↓*** (q<0.001)
Pnl MTPS4	β-pinene	0.579	0.020	<0.001	ns (q=0.579)	↑*** (q<0.001)	↑*** (q<0.001)	↑*** (q<0.001)
Pnl MTPS5	α-pinene	0.041	<0.001	<0.001	↓* (q=0.041)	↓* (q=0.012)	↑*** (q<0.001)	↑*** (q<0.001)
Pnl MTPS6	(-)-β-phellandrene	0.027	<0.001	<0.001	↓* (q=0.027)	↓*** (q<0.001)	↓** (q=0.005)	↑*** (q<0.001)
Pnl MTPS7	α-pinene	0.438	<0.001	<0.001	ns (q=0.438)	↓** (q=0.009)	ns (q=0.374)	↑*** (q<0.001)

Note: Linear models were fitted separately for each monoterpenoid using log2-transformed concentrations as the response variable and Treatment, Time and Treatment × Time as fixed effects. Reported *p* values refer to Type III ANOVA tests. Time-specific contrasts compare PPM-infested plants with non-infested controls at the same sampling time. *q* values were obtained by Benjamini–Hochberg correction across the four contrasts within each monoterpenoid. Arrows indicate higher (↑) or lower (↓) values in PPM-infested plants relative to controls; ns, not significant. Significance codes are based on *q* values: * $q < 0.05$; ** $q < 0.01$; *** $q < 0.001$. Values plotted in Figures 3 and 5 are untransformed means ± standard error of the mean.

Table S5. Linear-model analysis and time-specific treatment contrasts for diterpene resin acid (DRA) concentrations [μg (g dry weight)$^{-1}$] in Calabrian pine needles during pine processionary moth (PPM) infestation. DPI, days post-infestation. *DTPS*, diterpene synthase gene.

Variable	Assignment	Treatment p	Time p	Treatment × Time p	0 DPI	21 DPI	84 DPI	195 DPI
palustric acid	Putatively associated with *Pnl DTPS1* (abietane-type DRA)	0.397	0.966	<0.001	ns (q=0.397)	ns (q=0.293)	↑*** (q<0.001)	↑*** (q<0.001)
levopimaric acid	Putatively associated with *Pnl DTPS1* (abietane-type DRA)	0.133	0.735	<0.001	ns (q=0.133)	ns (q=0.133)	↑*** (q<0.001)	↑*** (q<0.001)
abietic acid	Putatively associated with *Pnl DTPS1* (abietane-type DRA)	0.788	0.002	<0.001	ns (q=0.788)	ns (q=0.785)	↑*** (q<0.001)	↑*** (q<0.001)
neoabietic acid	Putatively associated with *Pnl DTPS1* (abietane-type DRA)	0.785	0.516	<0.001	ns (q=0.785)	ns (q=0.785)	↑*** (q<0.001)	↑*** (q<0.001)
sandaracopimaric acid	Putatively associated with *Pnl DTPS3* (pimarane-type DRA)	0.615	0.571	0.527	ns (q=0.922)	ns (q=0.426)	ns (q=0.922)	ns (q=0.922)
isopimaric acid	Putatively associated with *Pnl DTPS3* (pimarane-type DRA)	0.929	0.404	0.015	ns (q=0.929)	ns (q=0.884)	ns (q=0.310)	↓** (q=0.009)
pimaric acid	Putatively associated with *Pnl DTPS4* (pimarane-type DRA)	0.952	<0.001	<0.001	ns (q=0.952)	↓*** (q<0.001)	ns (q=0.619)	↓** (q=0.004)
dehydroabietic acid	No specific *Pnl DTPS* assignment	0.750	<0.001	<0.001	ns (q=0.750)	ns (q=0.642)	↑*** (q<0.001)	↑*** (q<0.001)
abietic acid dehydroisomer	No specific *Pnl DTPS* assignment	0.556	0.857	0.879	ns (q=0.556)	ns (q=0.365)	ns (q=0.365)	ns (q=0.493)

Note: Linear models were fitted separately for each monoterpenoid using log2-transformed concentrations as the response variable and Treatment, Time and Treatment × Time as fixed effects. Reported p values refer to Type III ANOVA tests. Time-specific contrasts compare PPM-infested plants with non-infested controls at the same sampling time. q values were obtained by Benjamini–Hochberg correction across the four contrasts within each monoterpenoid. Arrows indicate higher (↑) or lower (↓) values in PPM-infested plants relative to controls; ns, not significant. Significance codes are based on q values: * $q < 0.05$; ** $q < 0.01$; *** $q < 0.001$. Values plotted in Figures 6 and 8 are untransformed means ± standard error of the mean.

Table S6. Linear-model analysis and time-specific treatment contrasts for diterpene synthase (*DTPS*) transcript abundances (relative expression) in Calabrian pine needles during pine processionary moth (PPM) infestation. DPI, days post-infestation; DRA, diterpene resin acid.

Variable	Assignment	Treatment p	Time p	Treatment × Time p	0 DPI	21 DPI	84 DPI	195 DPI
Pnl DTPS1	abietane-type DRAs	0.753	0.014	<0.001	ns (q=0.753)	↑* (q=0.038)	↑*** (q<0.001)	↑*** (q<0.001)
Pnl DTPS2	No DRA assigned; product relationship unresolved	0.774	0.446	<0.001	ns (q=0.774)	↓*** (q<0.001)	↓*** (q<0.001)	↓*** (q<0.001)
Pnl DTPS3	isopimaric and sandaracopimaric acids	0.701	0.020	<0.001	ns (q=0.849)	ns (q=0.849)	ns (q=0.849)	↓*** (q<0.001)
Pnl DTPS4	pimaric acid	0.736	0.994	<0.001	ns (q=0.736)	↓*** (q<0.001)	↓*** (q<0.001)	↓*** (q<0.001)

Note: Linear models were fitted separately for each monoterpenoid using log2-transformed concentrations as the response variable and Treatment, Time and Treatment × Time as fixed effects. Reported p values refer to Type III ANOVA tests. Time-specific contrasts compare PPM-infested plants with non-infested controls at the same sampling time. q values were obtained by Benjamini–Hochberg correction across the four contrasts within each monoterpenoid. Arrows indicate higher (↑) or lower (↓) values in PPM-infested plants relative to controls; ns, not significant. Significance codes are based on q values: * $q < 0.05$; ** $q < 0.01$; *** $q < 0.001$. Values plotted in Figures 6 and 7 are untransformed means ± standard error of the mean.

Table S7. Principal component analysis (PCA) loadings for metabolite and terpene synthase (*TPS*) transcript datasets.

Dataset	Variable	PC1	PC2	PC3
**Metabolite profiles**	**Explained variance (%)**	**64.800**	**14.567**	**7.699**
**Metabolite profiles**	**Cumulative variance (%)**	**64.800**	**79.367**	**87.066**
Metabolite profiles	tricyclene	0.205	0.266	-0.078
Metabolite profiles	α-thujene	0.175	0.367	-0.084
Metabolite profiles	α-pinene	0.251	-0.064	0.066
Metabolite profiles	α-fenchene	0.247	0.109	-0.057
Metabolite profiles	camphene	0.236	-0.071	0.141
Metabolite profiles	sabinene	0.239	-0.047	0.122
Metabolite profiles	β-pinene	0.249	-0.076	-0.060
Metabolite profiles	β-myrcene	0.247	0.014	0.133
Metabolite profiles	δ-3-carene	-0.193	0.169	0.238
Metabolite profiles	(-)-β-phellandrene	0.202	0.285	-0.178
Metabolite profiles	limonene	0.211	0.284	-0.090
Metabolite profiles	terpinolene	0.238	0.160	-0.025
Metabolite profiles	α-terpineol	0.237	0.025	-0.010
Metabolite profiles	bornyl acetate	0.243	0.093	0.024
Metabolite profiles	pimaric acid	-0.039	0.340	0.447
Metabolite profiles	sandaracopimaric acid	-0.012	0.134	0.538
Metabolite profiles	isopimaric acid	-0.089	-0.304	0.353
Metabolite profiles	palustric acid	0.244	-0.158	0.109
Metabolite profiles	levopimaric acid	0.230	-0.211	0.154
Metabolite profiles	dehydroabietic acid	0.206	-0.281	0.090
Metabolite profiles	abietic acid	0.220	-0.238	0.139
Metabolite profiles	neoabietic acid	0.241	-0.160	0.061
Metabolite profiles	abietic acid dehydroisomer	0.050	-0.289	-0.382
**TPS transcript profiles**	**Explained variance (%)**	**66.815**	**18.662**	**7.136**
**TPS transcript profiles**	**Cumulative variance (%)**	**66.815**	**85.477**	**92.613**
TPS transcript profiles	*Pnl MBOS1*	0.337	-0.253	0.039
TPS transcript profiles	*Pnl MTPS2*	0.362	0.085	-0.072
TPS transcript profiles	*Pnl MTPS3*	-0.337	0.229	0.205
TPS transcript profiles	*Pnl MTPS4*	0.359	-0.014	0.119
TPS transcript profiles	*Pnl MTPS5*	0.326	0.226	0.228
TPS transcript profiles	*Pnl MTPS6*	0.222	0.497	-0.009
TPS transcript profiles	*Pnl MTPS7*	0.194	0.492	0.315
TPS transcript profiles	*Pnl DTPS1*	0.335	0.078	0.133
TPS transcript profiles	*Pnl DTPS2*	-0.314	0.347	-0.070
TPS transcript profiles	*Pnl DTPS3*	-0.193	-0.235	0.873
TPS transcript profiles	*Pnl DTPS4*	-0.267	0.393	-0.030

Note: PCA was performed on log2-transformed and autoscaled matrices. Values represent loadings of each variable on the first three principal components; larger absolute values indicate stronger contributions to the corresponding component. Variance rows report the percentage of total variance explained by each principal component and the cumulative variance explained. PC, principal component.

Table S8. Factorial permutational multivariate analysis of variance (PERMANOVA) results and time-specific PERMANOVA contrasts for metabolite and terpene synthase (TPS) transcript profiles. df, degrees of freedom; DPI, days post-infestation; PPM, pine processionary moth; R^2, effect size; SS, sum of squares.

Dataset	Analysis	Effect / contrast	Time (DPI)	df	Residual df	SS	R^2	Pseudo-F	p	q	Permutations
Metabolite profiles	Factorial PERMANOVA	Treatment	—	1	16	138.963	0.252	32.913	<0.001	<0.001	9999
Metabolite profiles	Factorial PERMANOVA	Time	—	3	16	169.906	0.308	13.414	<0.001	<0.001	9999
Metabolite profiles	Factorial PERMANOVA	Treatment × Time	—	3	16	175.576	0.318	13.862	<0.001	<0.001	9999
TPS transcript profiles	Factorial PERMANOVA	Treatment	—	1	16	101.543	0.385	211.600	<0.001	<0.001	9999
TPS transcript profiles	Factorial PERMANOVA	Time	—	3	16	54.947	0.208	38.168	<0.001	<0.001	9999
TPS transcript profiles	Factorial PERMANOVA	Treatment × Time	—	3	16	99.832	0.378	69.345	<0.001	<0.001	9999
Metabolite profiles	Time-specific PERMANOVA	PPM-infested vs Control	0	1	4	2.004	0.141	0.654	0.700	0.700	20
Metabolite profiles	Time-specific PERMANOVA	PPM-infested vs Control	21	1	4	19.462	0.479	3.684	0.100	0.133	20
Metabolite profiles	Time-specific PERMANOVA	PPM-infested vs Control	84	1	4	103.386	0.871	26.893	0.100	0.133	20
Metabolite profiles	Time-specific PERMANOVA	PPM-infested vs Control	195	1	4	189.688	0.910	40.369	0.100	0.133	20
TPS transcript profiles	Time-specific PERMANOVA	PPM-infested vs Control	0	1	4	0.596	0.201	1.009	0.600	0.600	20
TPS transcript profiles	Time-specific PERMANOVA	PPM-infested vs Control	21	1	4	39.343	0.935	57.618	0.100	0.133	20
TPS transcript profiles	Time-specific PERMANOVA	PPM-infested vs Control	84	1	4	48.750	0.961	97.357	0.100	0.133	20
TPS transcript profiles	Time-specific PERMANOVA	PPM-infested vs Control	195	1	4	112.686	0.995	775.678	0.100	0.133	20

Note: Factorial PERMANOVA was performed on Euclidean distance matrices calculated from log2-transformed and autoscaled metabolite or *TPS* transcript data, as summarized in Table 2. Factorial terms were fitted sequentially in the order shown. Time-specific PERMANOVA contrasts compare PPM-infested plants with non-infested controls at each sampling time. p values were obtained from permutation tests, and q values were obtained by Benjamini–Hochberg correction within each analysis family. Exact condition-label permutations were used for time-specific contrasts; with n = 3 plant-level biological samples per experimental condition and time point, only 20 unique label allocations were possible, limiting the resolution of exact p values.

Table S9. Pearson correlations between terpene synthase transcript abundances and putatively associated terpenoid products or diterpene resin acids (DRA). *DTPS*, diterpene synthase gene; *MTPS*, monoterpene synthase gene; ns, not significant.

Gene	Putatively associated metabolite/product	Class	Association type	N	r	p	q	Significance
Pnl MTPS2	α-terpineol	Monoterpenoid	product-level	24	0.890	<0.001	<0.001	***
Pnl MTPS3	δ-3-carene	Monoterpenoid	product-level	24	0.970	<0.001	<0.001	***
Pnl MTPS4	β-pinene	Monoterpenoid	product-level	24	0.922	<0.001	<0.001	***
Pnl MTPS5	α-pinene	Monoterpenoid	product-level	24	0.924	<0.001	<0.001	***
Pnl MTPS6	(-)-β-phellandrene	Monoterpenoid	product-level	24	0.754	<0.001	<0.001	***
Pnl MTPS7	α-pinene	Monoterpenoid	product-level	24	0.477	0.018	0.020	*
Pnl DTPS1	palustric acid	DRA	pathway-level	24	0.973	<0.001	<0.001	***
Pnl DTPS1	levopimaric acid	DRA	pathway-level	24	0.939	<0.001	<0.001	***
Pnl DTPS1	abietic acid	DRA	pathway-level	24	0.909	<0.001	<0.001	***
Pnl DTPS1	neoabietic acid	DRA	pathway-level	24	0.956	<0.001	<0.001	***
Pnl DTPS3	sandaracopimaric acid	DRA	pathway-level	24	0.057	0.790	0.790	ns
Pnl DTPS3	isopimaric acid	DRA	pathway-level	24	0.515	0.010	0.012	*
Pnl DTPS4	pimaric acid	DRA	pathway-level	24	0.538	0.007	0.009	**

Note: Pearson correlation coefficients were calculated using log2-transformed values across all 24 plant-level biological samples. Only transcript–metabolite associations defined a priori in Table 1 were tested. q values were obtained by Benjamini–Hochberg correction across all correlations summarized in the table. Significance codes are based on q values: * $q < 0.05$; ** $q < 0.01$; *** $q < 0.001$.

Table S10. Primer pairs used for qRT-PCR analysis of terpene synthase genes and reference genes in Calabrian pine (*Pinus nigra* subsp. *laricio*) needles.

Gene	Forward primer (5′–3′)	Reverse primer (5′–3′)
MBOS1	GCAATGTTCCAATCACTTCCAAG	AGGCACAGGCTCAATGACG
MTPS2	GCTTCTCAAACCAGACTGC	TTGGTGGCGACGCTGTAG
MTPS3	TCTCGGGTCAACACAGGAAG	CCATCTCGGTAGTTGTAGAAGTG
MTPS4	CTGGAGCAACAGAGGAAGATG	GTATTTGTAACCGTAATGGAAAGC
MTPS5	GCCCGTGGAGAAGAAGC	TGGGAACGCTGCTGTTTG
MTPS6	TTAAACCCGACAGCAATGTTCCC	GTGGCATCACCGTAACCATCTC
MTPS7	TGCTGTCAATCAAGTCAATGC	GCAACACTGAAGCCATCTCTG
DTPS1	CGACACTTGCAGGAGATTG	TATGTGAAAGTGTCAAACCGTC
DTPS2	ACAGTACGTGCCGAACAA	AACCGGTTCGAAGAGGAC
DTPS3	AAAGATGTGCCGGAGAAGT	AGGATCTTCTTGACGTGTTGT
DTPS4	ATGCGAGATCAATGCAGC	ACGCAACTGGTTCGAACA
CYP	TGTAGAGGGCTTGGAGGTC	CAAGCGAGCTGTCCAGAGT
upLOC	GGTTTGCTTTGGAGGATATG	GTCCAATGTGCACCTCGT

Note: Primer sequences are reported in the 5′–3′ orientation. CYP, Cyclophilin; qRT-PCR, quantitative real-time PCR; upLOC, uncharacterized protein LOC103705956.
